# Reaction-free and MMP-independent fluorescent probes for long-term mitochondria visualization and tracking†
†Electronic supplementary information (ESI) available: Materials and methods; synthetic details, NMR spectra and HRMS spectra; photophysical data; and imaging data. CCDC 1851638 and 1851639. For ESI and crystallographic data in CIF or other electronic format see DOI: 10.1039/c8sc05119d


**DOI:** 10.1039/c8sc05119d

**Published:** 2018-12-11

**Authors:** Ruoyao Zhang, Guangle Niu, Xuechen Li, Lifang Guo, Huamiao Zhang, Rui Yang, Yuncong Chen, Xiaoqiang Yu, Ben Zhong Tang

**Affiliations:** a Center of Bio and Micro/Nano Functional Materials , State Key Laboratory of Crystal Materials , Shandong University , Jinan 250100 , China . Email: yuxq@sdu.edu.cn; b Department of Chemistry , Hong Kong Branch of Chinese National Engineering Research Center for Tissue Resto-ration and Reconstruction , Institute for Advanced Study , Division of Biomedical Engineering and Division of Life Science , The Hong Kong University of Science and Technology , Clear Water Bay , Kowloon , Hong Kong 999077 , China . Email: tangbenz@ust.hk

## Abstract

Visualizing and tracking mitochondrial dynamic changes is crucially important in the fields of physiology, pathology and pharmacology. Traditional electrostatic-attraction based mitochondrial probes fail to visualize and track the changes due to their leakage from mitochondria when mitochondrial membrane potential (MMP) decreases. Reaction-based MitoTracker probes can realize visualization and tracking of mitochondria changes independent of MMP changes. However, such probes impair mitochondrial proteins and exhibit high cytotoxicity. Therefore, it still remains challenging to explore reaction-free and highly biocompatible probes for visualizing and tracking mitochondrial dynamics independent of MMP fluctuations. Herein we synthesized two reaction-free fluorescent mitochondrial probes ECPI-12 and IVPI-12 bearing a long C_12_-alkyl chain. These cationic probes can firmly immobilize in the mitochondrial inner membrane by strong hydrophobic interaction between the C_12_-alkyl chain and lipid bilayer, resulting in high specificity and long-term mitochondrial staining regardless of MMP changes. They also exhibit large two-photon absorption cross-sections and show deep penetration in live tissues in two-photon microscopy. Furthermore, they display excellent biocompatibility and realize *in situ* and real-time mitophagy tracking in live cells. These excellent properties could make ECPI-12 and IVPI-12 the first selective tools for long-term visualization and tracking of mitochondrial dynamics.

## Abstract 2

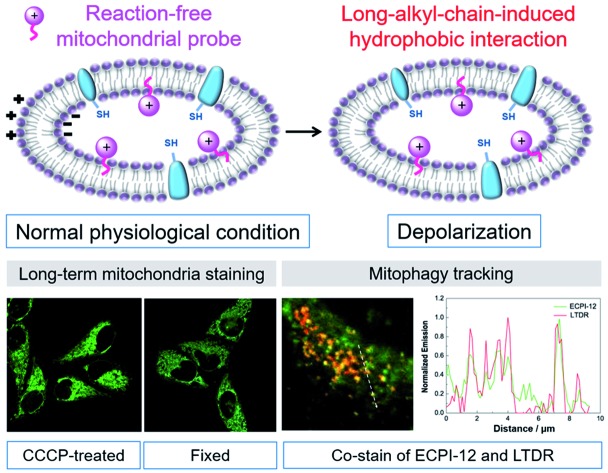
We present two mitochondria-immobilized fluorescent probes ECPI-12 and IVPI-12 for long-term mitochondria visualization and tracking regardless of MMP changes.

## Introduction

The dynamic changes of mitochondrial morphology and number are highly relevant to numerous physiological and pathological processes.^1^,^2^ Recent studies have indicated that mitochondrial morphology changes from a tubular network to a punctiform and fragmented formation during the early stage of apoptotic cell death.^3^ Researchers also found that damaged or unwanted mitochondria can be selectively removed during the mitophagy process to maintain a healthy population of mitochondria.^4^–^7^ In addition, mitochondrial dynamics also play indispensable roles in controlling antitumor immune responses.^8^ Therefore, visualizing and tracking mitochondrial dynamic changes is crucially important in the fields of physiology, pathology and pharmacology.

**Scheme 1:**
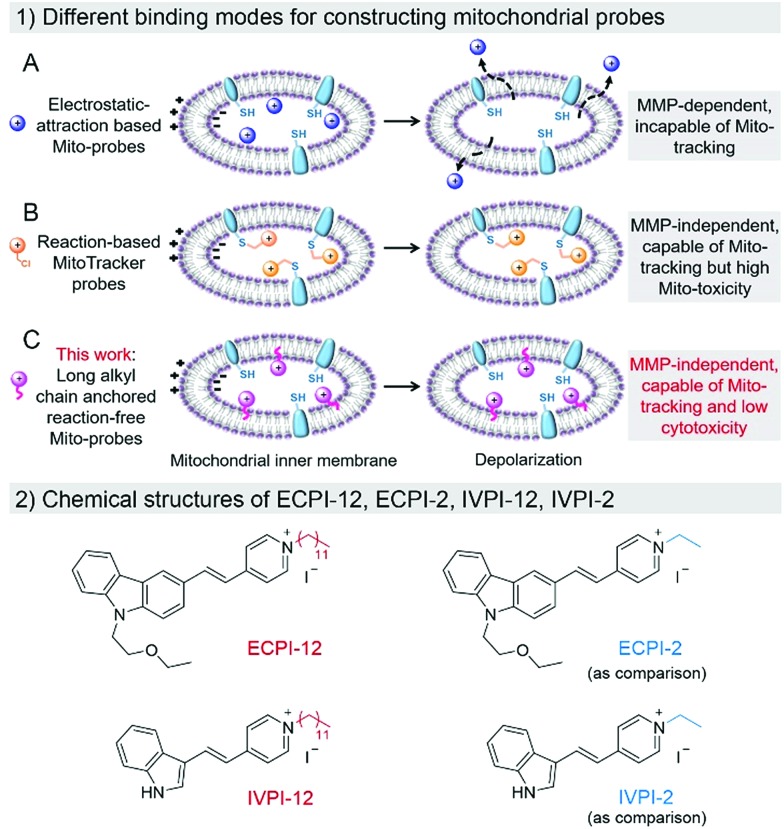
(1) Different binding modes for constructing electrostatic-attraction based Mito-probes (A), reaction-based MitoTracker probes (B), and long alkyl chain anchored reaction-free Mito-probes (C) within the mitochondrial inner membrane in normal and depolarized mitochondria. (2) Chemical structures of ECPI-12, ECPI-2, IVPI-12, and IVPI-2.

Fluorescence microscopy has become a powerful tool for real-time visualization and tracking of biomolecules in medical and biological applications,^9^–^18^ due to its high selectivity, remarkable sensitivity, excellent spatial and temporal resolution and non-invasive operation.^19^–^25^ Current fluorescent probes for visualizing and tracking mitochondria associated dynamic changes, such as monitoring the mitophagy process, could be divided into two classes: traditional electrostatic-attraction based cationic mitochondrial probes and reaction-based MitoTracker probes (Scheme 1(1)). Traditional electrostatic-attraction based cationic mitochondrial probes like rhodamine 123 and most of the mitochondrial probes reported in the literature target mitochondria with high dependence on the large negative MMP.^26^–^29^ Once the MMP decreases or vanishes, these probes will leak from mitochondria (Scheme 1(1A)), limiting their use in tracking mitochondria with fluctuating MMP in biological events.

To solve the above-mentioned problem, reaction-based MitoTracker probes have been developed, such as commercial MitoTracker Red and MitoTracker Deep Red.^30^–^32^ Besides cationic properties, these kinds of mitochondrial probes contain an additional reactive moiety like the benzyl chloride group. The cationic properties first drive these probes into mitochondria *via* electrostatic interaction, and the benzyl chloride group can react with nucleophiles, such as the thiol group of peptides and proteins in mitochondria, resulting in high immobilization in mitochondria even if the MMP decreases or vanishes (Scheme 1(1B)). However, such probes would inevitably consume the thiol group of mitochondrial proteins, and cause perturbation in the normal metabolism in mitochondria, resulting in high cytotoxicity to live cells.^33^–^35^ Therefore, development of biocompatible mitochondrial probes based on a reaction-free strategy for mitochondria visualization and tracking remains crucially important, and is still extremely challenging.

Generally, cell plasma membrane probes like DiI and DiO possess long lipophilic aliphatic chains.^36^–^38^ These probes can stain the plasma membrane in fixed cells, only depending on hydrophobic interaction between the long lipophilic aliphatic chains and the lipid bilayer of the membrane. Inspired by this design, we anticipate that a long-lipophilic-aliphatic-chain-modified cationic probe could realize long-term retention in mitochondria by the above-mentioned strong hydrophobic interaction, which is probably independent of the influence of MMP changes (Scheme 1(1C)). On the other hand, such reaction-free mitochondrial probes could show good biocompatibility in live cells and be beneficial for long-term mitochondrial dynamic tracking.

In this work, two mitochondrial probes ECPI-12 and IVPI-12 modified with a C_12_-alkyl chain were rationally designed and synthesized based on carbazole- and indole-pyridinium, respectively (Scheme 1(2)). These two probes could target mitochondria by means of electrostatic interaction, and firmly locate in mitochondria by strong hydrophobic interaction regardless of the fluctuation of MMP. By comparing with ECPI-2 and IVPI-2 bearing a short alkyl chain (Scheme 1(2)), we proved that the long-alkyl-chain-induced hydrophobic interaction allows ECPI-12 and IVPI-12 to be retained for a long time in mitochondria when the MMP decreases or vanishes. These reaction-free probes show lower cytotoxicity than the reaction-based commercial probe MitoTracker Deep Red FM (MTDR). Their two-photon imaging performance was also evaluated in live cells and tissues. In addition, these two probes could successfully track mitophagy in live cells.

## Results and discussion

### Design and synthesis

**Fig. 1:**
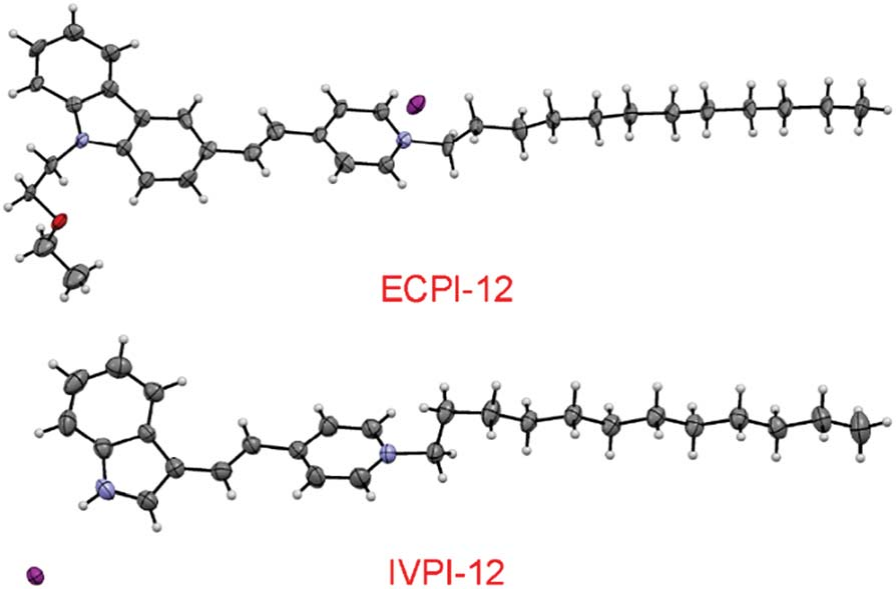
Single-molecular configurations of ECPI-12 and IVPI-12 in the respective single crystal cell with atoms labeled in color. C, gray; H, white; N, blue; O, red; I, purple.

To realize the stable immobilization of fluorescent probes in mitochondria by non-covalent interaction, we anticipated that decorating a long alkyl chain on the positively charged fluorescent probes is probably an effective and reaction-free strategy. On the other hand, carbazole- and indole-pyridinium were chosen as the fluorophores due to their simple synthesis, excellent membrane permeability, low toxicity and good photostability.^39^,^40^ Thus, a long C_12_-alkyl chain was added on the pyridinium side to construct reaction-free probes ECPI-12 and IVPI-12 for long-term mitochondria staining and tracking by strong hydrophobic interaction. For comparison, ECPI-2 and IVPI-2 were also synthesized by adding a short C_2_-alkyl chain on the pyridinium side. The synthetic routes to ECPI-12, IVPI-12, ECPI-2, and IVPI-2 are depicted in Scheme S1.† The chemical structures of these molecules were fully characterized by ^1^H NMR, ^13^C NMR, and HRMS (Fig. S1–S16†). In addition, the structures of ECPI-12 and IVPI-12 were further confirmed by single-crystal X-ray diffraction analysis (CCDC ; 1851638 and ; 1851639, Fig. 1). The details of the experimental conditions, unit cell data and refinement data are summarized in Tables S1 and S2.†

### Photophysical properties

**Fig. 2:**
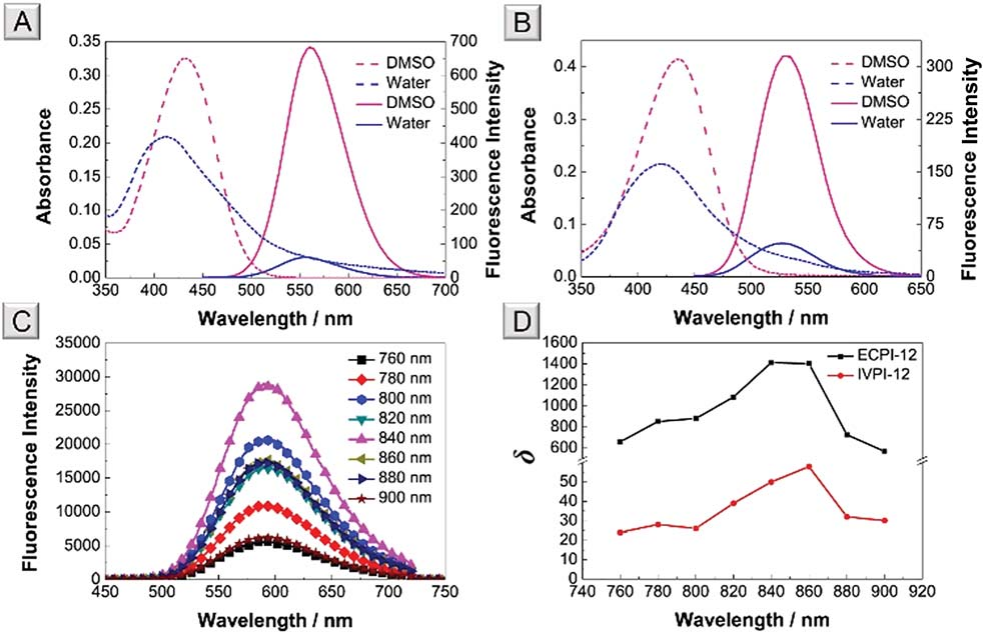
Absorption (dashed line) and OPEF spectra (solid line) of ECPI-12 (A) and IVPI-12 (B) in DMSO and water. (C) TPEF spectra of ECPI-12 in DMSO excited at 760, 780, 800, 820, 840, 860, 880, and 900 nm, respectively. (D) Two-photon absorption cross-section (*δ*) of ECPI-12 and IVPI-12 in DMSO. Concentration: 10 μM.

The absorption and one-photon excitation fluorescence (OPEF) spectra of ECPI-12 and IVPI-12 in DMSO and water are given in Fig. 2A and B, and their corresponding photophysical data are summarized in Table S3.† ECPI-12 exhibited slightly blue-shifted absorption compared to IVPI-12 in DMSO and water (Fig. 2A, B and Table S3†). However, ECPI-12 showed red-shifted emission (greenish yellow fluorescence) compared to IVPI-12 (green fluorescence), probably due to larger π-conjugation. ECPI-12/IVPI-12 showed obviously higher fluorescence quantum yield (*Φ*) in DMSO than that in aqueous solution (Table S3†). The low quantum yield under aqueous conditions is probably attributed to the aggregation caused quenching effect,^41^ which could efficiently reduce the background signals from the probes distributed in the cytoplasm or cell culture medium. Likewise, the absorption and fluorescence spectra of ECPI-2 and IVPI-2 in different solvents were also measured and are plotted in Fig. S17.† These two probes with short alkyl chains display similar optical properties to ECPI-12 and IVPI-12 (Table S3†).

Generally, organic dyes with a donor–acceptor structure exhibit good two-photon absorption (TPA) and two-photon excitation fluorescence (TPEF).^42^,^43^ The TPEF spectra of ECPI-12 and IVPI-12 excited at different pulse wavelengths (760–900 nm) in DMSO were investigated. ECPI-12 and IVPI-12 showed high TPEF in DMSO (Fig. 2C and S18†). Using fluorescein as the standard,^44^ the two-photon absorption cross-section (*δ*) of ECPI-12 and IVPI-12 was calculated to be 1412 GM and 58 GM (Fig. 2D and Table S4†), excited at 840 nm and 860 nm, respectively. Such high *δ* values are beneficial for two-photon imaging in live cells and deep tissues.

### *In vitro* one-photon fluorescence imaging

**Fig. 3:**
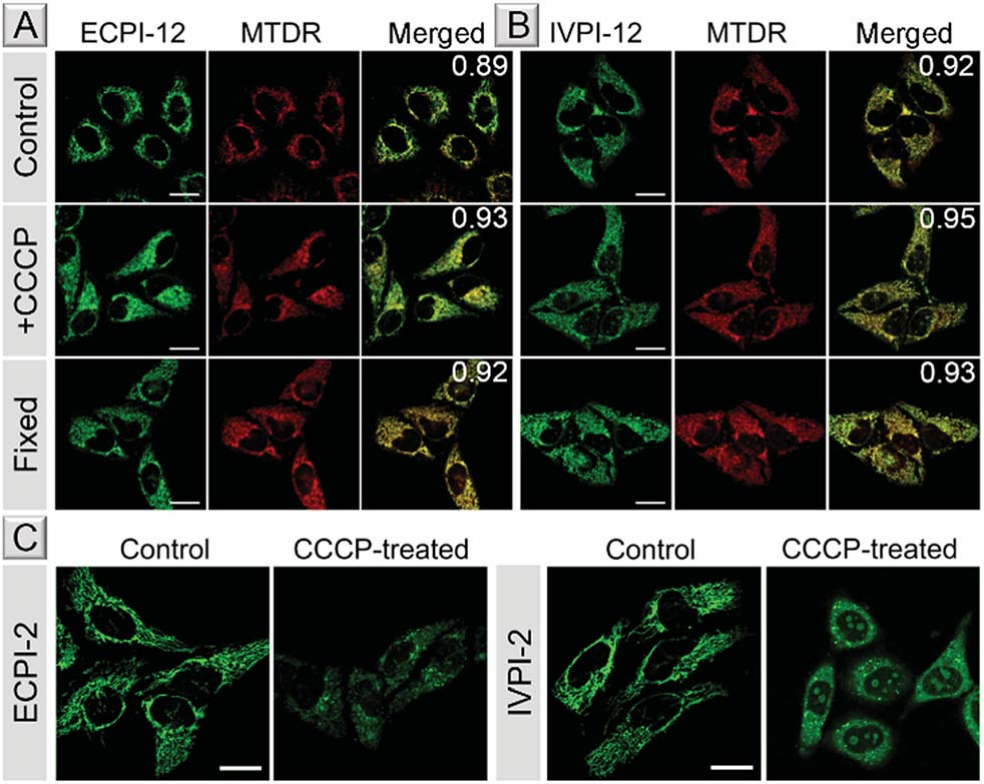
CLSM images of live, CCCP-treated and fixed HeLa cells stained with 0.2 μM ECPI-12 (A) or IVPI-12 (B) and 0.2 μM MTDR. Co-localization coefficients of ECPI-12 or IVPI-12 and MTDR are shown in the merged images. (C) CLSM images of live HeLa cells stained with 0.2 μM ECPI-2 and 0.2 μM IVPI-2 treated with or without 15 μM CCCP, respectively. Scale bar = 20 μm.

The bioimaging properties of ECPI-12 and IVPI-12 in live cells were investigated by confocal laser scanning microscopy (CLSM). After incubation in live HeLa cells at different concentrations (0.1 μM, 0.2 μM and 0.5 μM) for 30 min, bright fluorescence of ECPI-12 and IVPI-12 from the filamentous structures in the cytoplasm could be collected at a low concentration of 0.1 μM (Fig. S19†), which is the typical morphology of mitochondria.^45^ The fluorescence intensity of ECPI-12 and IVPI-12 in HeLa cells was enhanced with increased incubation concentration. Then we investigated the uptake efficiency of ECPI-12 and IVPI-12 in live cells at different time points. The corresponding fluorescent images and mean fluorescence intensities revealed that ECPI-12 and IVPI-12 reached the maximum uptake within 30 min (Fig. S20–S22†). After 30 min incubation, reconstructed 3D fluorescent images were obtained and basically no fluorescence could be seen out of the cells (Fig. S23†), confirming their excellent cell permeability. A co-staining experiment with a commercial mitochondrial probe, MTDR, was carried out to further confirm their location in mitochondria. As shown in Fig. 3A and B, the fluorescence signal of ECPI-12/IVPI-12 displayed an excellent overlap with that of MTDR. The co-localization coefficients for ECPI-12/IVPI-12 and MTDR were 0.89 and 0.92, respectively. Another cell line, A549, was also used to test the imaging performance, and the data further showed that ECPI-12 and IVPI-12 can effectively locate in mitochondria (Fig. S24 and S25†). These data indicated that ECPI-12 and IVPI-12 exhibited remarkable cell permeability and specifically stain mitochondria in live cells at a low concentration of 0.2 μM.

MMP is a key parameter for mitochondrial functions, and the decrease or vanishing of MMP will result in cellular dysfunction or even death.^46^,^47^ To assess whether ECPI-12 and IVPI-12 could still stain mitochondria when MMP decreases, live HeLa cells were pretreated with ECPI-12/IVPI-12 and MTDR, and then treated with CCCP. CCCP is a type of protonophore and can cause rapid acidification of mitochondria by collapsing MMP.^48^ MTDR is a reaction-based mitochondrion-selective probe that can be well retained in mitochondria regardless of MMP changes. Fig. 3A and B show that the staining patterns of ECPI-12/IVPI-12 and MTDR were still well overlapped with the co-localization coefficients of 0.93 and 0.95, respectively. The results revealed that ECPI-12 and IVPI-12 can firmly locate in mitochondria regardless of the decrease of MMP.

To further verify the excellent staining ability of ECPI-12 and IVPI-12 in mitochondria when MMP vanishes, live HeLa cells stained with ECPI-12/IVPI-12 and MTDR were fixed with 4% paraformaldehyde for 30 min. As shown in Fig. 3A and B, the staining pattern of ECPI-12/IVPI-12 displayed a good overlap with that of MTDR. In addition, the co-localization coefficients of ECPI-12/IVPI-12 and MTDR were 0.92 and 0.93, respectively, demonstrating that ECPI-12 and IVPI-12 can still stain mitochondria exclusively when MMP vanishes.

### Mechanism study

Why do ECPI-12 and IVPI-12 show excellent retention in mitochondria when MMP decreases or vanishes? We anticipated that the long alkyl chain played a crucial role in such a function. To prove that, corresponding probes ECPI-2 and IVPI-2 with a short alkyl chain were applied for live cell imaging for comparison. We could see that ECPI-2 and IVPI-2 could also stain filamentous structures in the cytoplasm of live cells *via* electrostatic interaction (Fig. 3C). The staining regions for ECPI-2 and IVPI-2 in live HeLa cells were confirmed to be mitochondria by co-stain experiments with high co-localization coefficients (Fig. S26†). However, when MMP decreased upon treatment with CCCP, both ECPI-2 and IVPI-2 lost their staining patterns in filamentous structures of mitochondria (Fig. 3C). ECPI-2 showed staining of dispersed dots in the cytoplasm and a low overlap with MTDR (Fig. S27†). Interestingly, IVPI-2 diffused into the nucleus and stained the nucleolus directly after treatment with CCCP. These data demonstrated that ECPI-2 and IVPI-2 displayed low mitochondrial selectivity when MMP decreases. It should be noted that the differences between ECPI-12/IVPI-12 and ECPI-2/IVPI-2 are the length of the alkyl chain on the pyridinium side (Scheme 1(2)). Therefore, the long alkyl chain has a decisive role in making ECPI-12 and IVPI-12 exclusively locate in mitochondria regardless of the decrease of MMP. Considering the components of the mitochondrial inner membrane, the long-alkyl-chain-induced strong hydrophobic interaction with membrane phospholipids allows ECPI-12 and IVPI-12 to firmly stain mitochondria for a long time regardless of MMP changes.

To further confirm this stable hydrophobic interaction, live HeLa cells stained with ECPI-12/IVPI-12 and MTDR were fixed with 4% paraformaldehyde for 30 min, and placed at room temperature for 12 h. As shown in Fig. S28,† ECPI-12/IVPI-12 still showed a good overlap with MTDR, and the co-localization coefficients of ECPI-12/IVPI-12 and MTDR were 0.95 and 0.89, respectively, indicating that they can reside in mitochondria for a long time after MMP vanished. What's more, further imaging experiments in live A549 cells were also carried out to verify the mitochondria-staining performance of ECPI-12/IVPI-12 under the conditions of MMP changes (Fig. S25†). Taken together, all these results confirmed that the hydrophobic interaction between the long alkyl chain of ECPI-12/IVPI-12 and phospholipids in the mitochondrial inner membrane is extremely stable.

### *In vitro* and *ex vivo* two-photon imaging

**Fig. 4:**
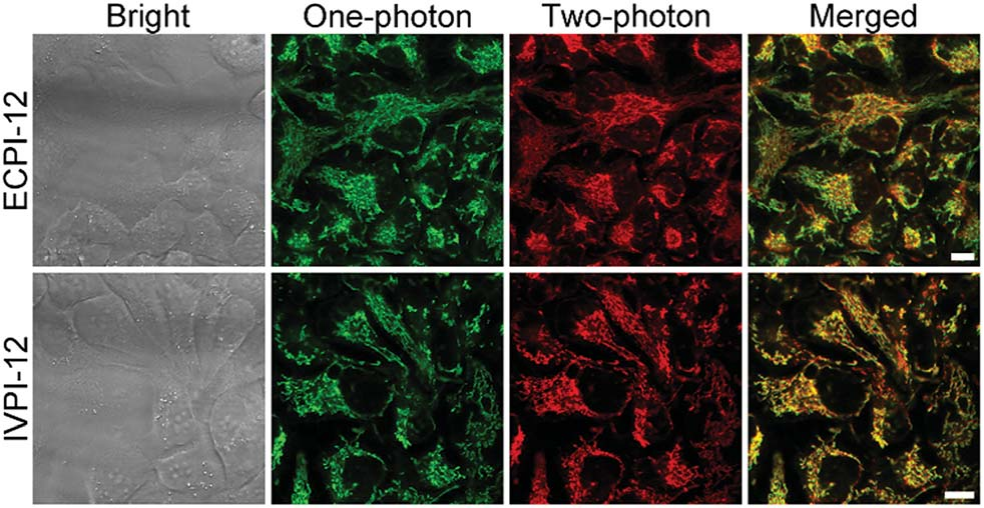
*In vitro* two-photon microscopy images of live HeLa cells stained with 0.2 μM ECPI-12 and 0.2 μM IVPI-12. One-photon excitation: 488 nm. Two-photon excitation: 840 nm for ECPI-12 and 860 nm for IVPI-12. Scale bar = 10 μm.

Given the high two-photon absorption cross-section values of ECPI-12 and IVPI-12 excited at 840 nm and 860 nm, respectively, we first performed *in vitro* two-photon imaging of these two probes in live HeLa cells. As displayed in Fig. 4, bright two-photon fluorescence with high signal-to-noise ratio from filamentous structures of mitochondria in the cytoplasm could be clearly collected, and these two-photon excitation fluorescence images showed similar distributions to those of one-photon excitation (Fig. 4), demonstrating the great potential of the probes in two-photon imaging.

**Fig. 5:**
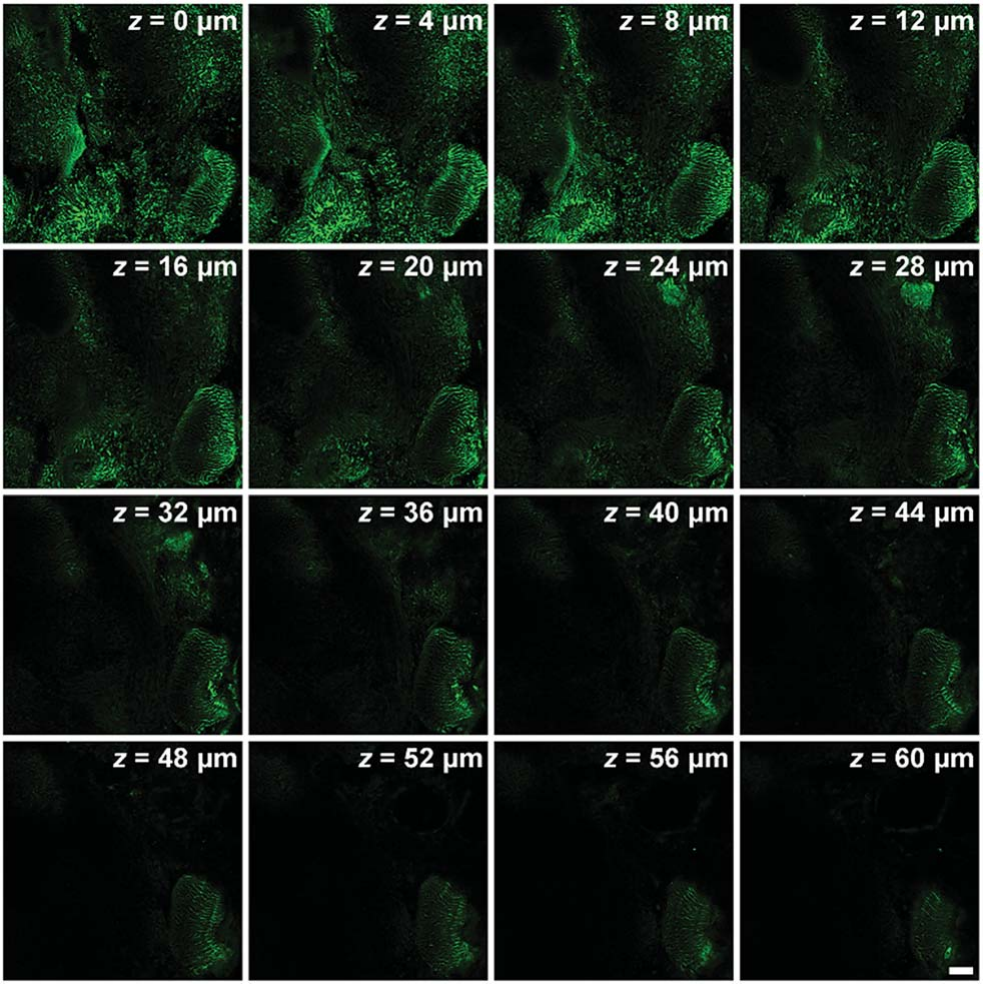
*Ex vivo* two-photon (*λ*_ex_ = 840 nm) microscopy images of mouse skeletal muscle tissue stained with 0.2 μM ECPI-12 at different penetration depths along the *Z* axis. Scale bar = 20 μm.

Two-photon microscopy outperforms one-photon microscopy in terms of low photodamage and photobleaching, high spatial resolution and deep tissue penetration.^49^–^54^ Then we evaluated the two-photon imaging performance of ECPI-12 and IVPI-12 in live rat skeletal muscle tissues. Two-photon fluorescence imaging data in Fig. S29† showed that mitochondria were regularly arranged and formed a reticulum, while the transverse plane showed a tubular morphology, which is consistent with the mitochondrial morphology in skeletal muscle obtained by scanning electron microscopy.^55^,^56^ Moreover, a series of two-photon fluorescent images were captured every 4 μm along the *Z* axis (Fig. 5 and S30†). The two-photon fluorescence signals with high contrast could be clearly detected at a depth of up to 60 μm and 52 μm for ECPI-12 and IVPI-12, respectively. These data indicated that ECPI-12 and IVPI-12 exhibit excellent permeability and high signal-to-noise ratio in *in vitro* and *ex vivo* two-photon imaging.

### Mitophagy tracking

**Fig. 6:**
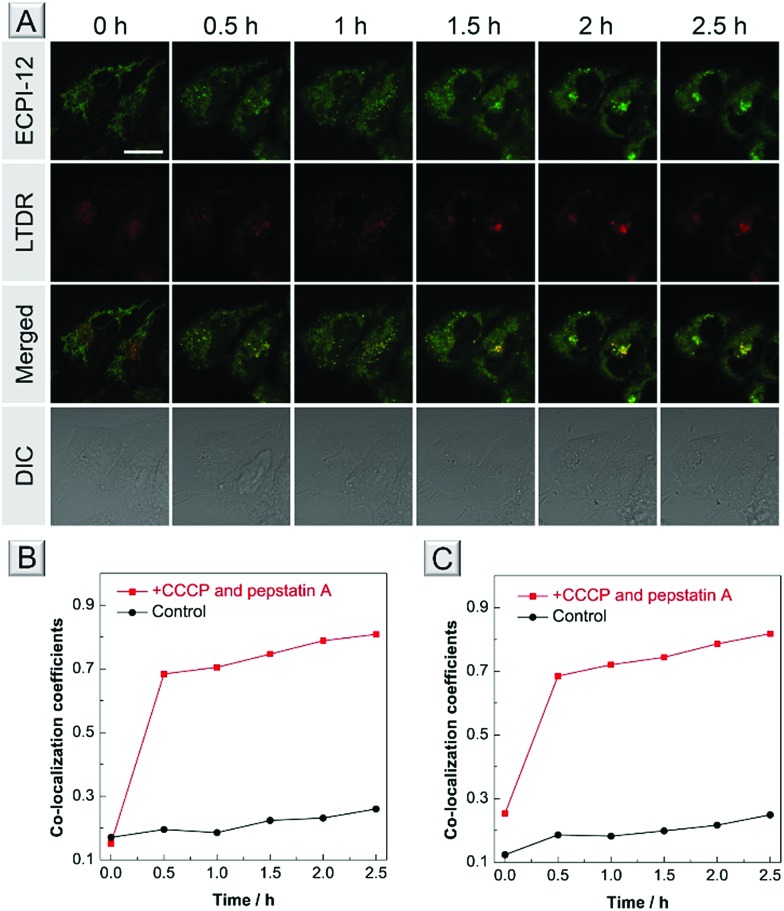
(A) Co-stained images of HeLa cells stained with 0.2 μM ECPI-12 and 0.2 μM LTDR after treatment with 10 μM CCCP and 7.5 μM pepstatin A at different time points. Scale bar = 20 μm. Co-localization coefficients of ECPI-12 (B) and IVPI-12 (C) with LTDR at different time points in live HeLa cells with or without CCCP and pepstatin A treatment.

A further bioimaging experiment was carried out to test whether they could track mitophagy. After staining with ECPI-12/IVPI-12 and lysosomal probe LysoTracker Deep Red (LTDR), HeLa cells were treated with 10 μM CCCP and 7.5 μM pepstatin A to induce mitophagy. We monitored the overlay changes of ECPI-12/IVPI-12 and LTDR by recording the co-localization coefficient during the mitophagy process. After treatment with CCCP and pep-statin A at different time points of 0 h, 0.5 h, 1 h, 1.5 h, 2 h, and 2.5 h, the fluorescent images were captured and the corresponding co-localization coefficient data were also recorded. The fluorescence signal of ECPI-12/IVPI-12 gradually overlapped with that of LTDR with an increase in time (Fig. 6A and S31†). And the co-localization coefficients of ECPI-12/IVPI-12 and LTDR increased from 0.15 to 0.81, and from 0.25 to 0.82, respectively (Fig. 6B, C and Table S5†). Such high overlay data successfully demonstrated the occurrence of mitophagy.^57^–^59^ In the control experiments without the treatment of CCCP and pepstatin A, however, the fluorescence of ECPI-12/IVPI-12 showed a very low overlap with that of LTDR after incubation for 2.5 h (Fig. S32 and S33†), which was directly revealed by the low co-localization coefficient values (Fig. 6B, C and Table S6†). Compared with other mitophagy probes,^6^,^7^,^57^–^59^ our probe could still monitor such a process when the MMP changes. The above-mentioned results confirmed that ECPI-12 and IVPI-12 can realize *in situ* and real-time mitophagy tracking in live cells.

### Cytotoxicity

**Fig. 7:**
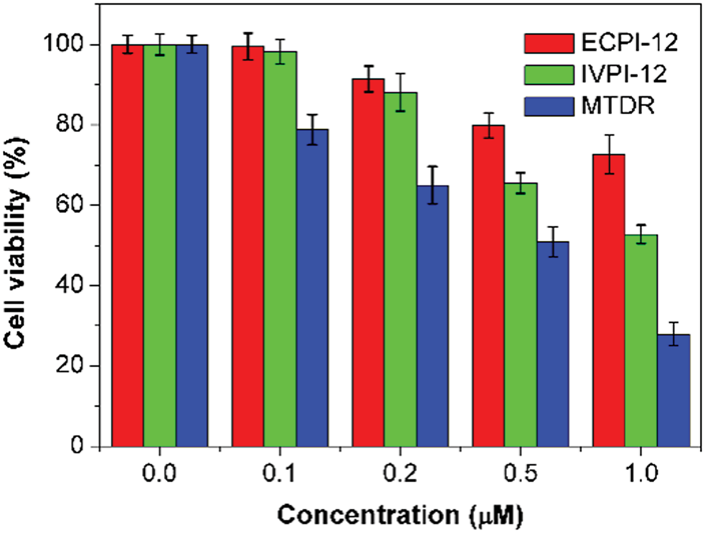
Viability of HeLa cells after incubation with ECPI-12, IVPI-12, and MTDR at different concentrations for 48 h.

The potential long-term cytotoxicity of bioprobes should be carefully considered for tracking and imaging in live cells. Then we studied the cytotoxicity of ECPI-12, IVPI-12, and MTDR to live HeLa cells by the standard MTT assay. It's clear to see that the viability of HeLa cells was higher than 80% after incubation with ECPI-12 and IVPI-12 at low concentration for 48 h (Fig. 7). However, the viability of HeLa cells incubated with MTDR was apparently lower than that of those incubated with ECPI-12 and IVPI-12 especially at increased concentration. These data indicated that the reaction-based mitochondrial probes like MTDR exhibited high cytotoxicity to live cells, while the reaction-free mitochondrial probes like ECPI-12 and IVPI-12 with a C_12_-alkyl chain exhibited low cytotoxicity and good biocompatibility.

## Conclusions

To summarize, we have successfully synthesized two probes, ECPI-12 and IVPI-12, bearing a C_12_-alkyl chain for mitochondria-immobilizing. Both ECPI-12 and IVPI-12 could exclusively target mitochondria in live cells, and particularly be immobilized in mitochondria regardless of MMP changes. Using ECPI-2 and IVPI-2 with a short alkyl chain for comparison, we prove that the long-alkyl-chain-induced strong hydrophobic interaction allows long-term retention of ECPI-12 and IVPI-12 in mitochondria when MMP decreases or vanishes. These two probes exhibit large values of two-photon absorption cross-section and can be used in live cells and live deep tissue imaging. Due to their long-term mitochondria staining capability, ECPI-12 and IVPI-12 can realize *in situ* and real-time mitophagy tracking in live cells. In addition, MTT data show that reaction-free probes ECPI-12 and IVPI-12 have good biocompatibility. These impressive results make ECPI-12 and IVPI-12 potential tools for monitoring and tracking the mitochondrial dynamic changes in live cells and live tissues. This work could further shed light on future design and synthesis of other biocompatible and functional probes for specific bioimaging applications in mitochondria and other subcellular organelles.

## Conflicts of interest

There are no conflicts to declare.

## Supplementary Material

Supplementary information

Crystal structure data
