# Supplementary material for: Reaction-free and MMP-independent fluorescent probes for long-term mitochondria visualization and tracking
Source: Chem Sci. 2018 Dec 11;10(7):1994–2000. doi: 10.1039/c8sc05119d (PMC6383331; doi:10.1039/c8sc05119d)
Supplement: Supplementary file 1 [file SC-010-C8SC05119D-s001.pdf]

Electronic Supplementary Material (ESI) for Chemical Science.
This journal is © The Royal Society of Chemistry 2018

Reaction-free and MMP-independent fluorescent probes for long-term mitochondria visualization and tracking

Ruoyao Zhang,[a,b,†] Guangle Niu,[b,†] Xuechen Li,[a] Lifang Guo,[a] Huamiao Zhang,[a] Rui Yang,[a] Yuncong Chen,[b] Xiaoqiang Yu,*[,a] and Ben Zhong Tang*[,b]

[a] Center of Bio and Micro/Nano Functional Materials, State Key Laboratory of Crystal Materials, Shandong University, Jinan 250100, China

[b] Department of Chemistry, Hong Kong Branch of Chinese National Engineering Research Center for Tissue Restoration and Reconstruction, Institute for Advanced Study, Division of Biomedical Engineering and Division of Life Science, The Hong Kong University of Science and Technology, Clear Water Bay, Kowloon, Hong Kong 999077, China

[†] These authors contributed equally to this work.

* yuxq@sdu.edu.cn; tangbenz@ust.hk

Materials and Methods

1.1 Apparatus and general methods

All the chemicals were purchased and used as received without further purification unless otherwise specified. The mitochondrial probe MitoTracker Deep Red FM (MTDR), lysosomal probe LysoTracker Deep Red (LTDR) were purchased from Molecular Probes. The UV-visible-near-IR absorption spectra of dilute solutions were recorded on a HITACH U-2910 spectrophotometer using a quartz cuvette with 1 cm path length. One-photon spectra were obtained on a HITACH F-2700 spectrafluorimeter equipped with a 450-W Xe lamp. Two-photon excited fluorescence (TPEF) spectra were measured on a SpectroPro300i, and the pump laser beam came from a mode-locked Ti: sapphire laser system with a pulse duration of 160 fs and a repetition rate of 76 MHz.

1.2 Measurement of fluorescence quantum yield and two-photon absorption cross section

Fluorescence quantum yield (Φ) can be calculated by means of Eq. (1):[1]

$$\Phi_s = \Phi_r \frac{A_r I(\lambda_r) n_s^2 F_s}{A_s I(\lambda_s) n_r^2 F_r} \quad (1)$$

s and r refer to the sample and the reference materials, respectively. Φ is the fluorescence quantum yield, F is the integrated emission intensity, A stands for the absorbance, and n is the refractive index. In this work, the quantum yields were calculated by using fluorescein (Φ = 0.95, pH = 13) as the standard.[2]

Two-photon absorption cross-section (δ) was measured using the two-photon induced fluorescence method, and thus the δ can be calculated by means of Eq. (2):[1]

$$\delta_s = \delta_r \frac{\Phi_r c_r n_r F_s}{\Phi_s c_s n_s F_r} \quad (2)$$

F is TPEF integral intensity. Φ is the fluorescence quantum yield. δ_r is the two-photon absorption cross-section of fluorescein in sodium hydroxide aqueous solution (pH = 13.0).[3]

1.3 Cell culture and staining, and tissue staining

Cell culture: HeLa and A549 cells were grown in Dulbecco's Modified Eagle Medium supplemented with 10% fetal bovine serum (FBS) and 1% penicillin and streptomycin in a 5% CO_2 incubator at 37 ^{o}C.

Cell staining experiment: ECPI-12 and IVPI-12 was dissolved in DMSO at a stock concentration of 0.5 mM, respectively. HeLa and A549 cells were placed on glass coverslips and allowed to adhere for 48 h. HeLa and A549 cells were incubated with probes in DMEM for 30 min at 37 ^{o}C.

Co-staining experiment: MTDR were dissolved in DMSO at a stock concentration of 0.1 mM. (1) In normal live cells: HeLa and A549 cells were firstly incubated with 0.2 μM MTDR for 30 min, then stained with 0.2 μM ECPI-12/IVPI-12 for 30 min; (2) In live cells with decreased mitochondrial membrane potential (MMP): HeLa and A549 cells were firstly incubated with 0.2 μM MTDR for 30 min, then stained with 0.2 μM ECPI-12/IVPI-12 for 30 min, and afterwards treated with 15 μM carbonyl cyanide *m*-chlorophenyl hydrazone (CCCP) for 20 min; (3) In fixed cells: HeLa and A549 cells were firstly incubated with 0.2 μM MTDR for 30 min, then stained with 0.2 μM ECPI-12/IVPI-12 for 30 min, and afterwards treated with 4% paraformaldehydefor 30 min. Every time the cells were washed with PBS to remove the unbound probe before stained with another probe.

Mitophagy tracking: We monitored the co-localization coefficient values of ECPI-12/IVPI-12 and LTDR during mitophagy process. HeLa cells were stained with 2 μM ECPI-12/IVPI-12 and 0.2 μM LTDR, and then treated with 10 μM CCCP and 7.5 μM pepstatin A to induce mitophagy. We recorded the fluorescent images at different treated time points of 0 h, 0.5 h, 1 h, 1.5 h, 2 h, 2.5 h, and obtained the corresponding co-localization coefficient.

Tissue staining: The rat skeletal muscle tissues were directly removed from just killed adult wistar rat (purchased from Laboratory Animal Center, Shandong University). Then the tissues were stained with ECPI-12/IVPI-12 (0.2 μM) at room temperature in H-DMEM supplemented with 10 % fetal bovine serum (FBS) and 1% penicillin and streptomycin for 1 h. The tissues were washed with PBS to remove the unbound probe before performing two-photon imaging.

1.4 Cell-viability assay

The study of the effect of ECPI-12/IVPI-12 on cell viability was carried out using the standard MTT assay. HeLa cells growing in log phase were seeded into 96-well plates (ca. 1×10^4 cells/well) and allowed to adhere for 24 h. ECPI-12/IVPI-12 and MTDR dissolved in DMEM at concentrations of 0.1 μM, 0.2 μM, 0.5 μM, and 1.0 μM, respectively, were added into the wells as the treatment group (200 μL/well), and DMEM without dyes was added into the wells as the negative control group. The cells were incubated for 48 h at 37 °C under 5% CO_2. Then MTT (5 mg/mL in DMEM) was added into each well. After 4 h incubation at 37 °C, 200 μL DMSO was added to dissolve the purple crystals. After 20 min incubation, the optical density readings at 570 nm were taken using a plate reader. Cytotoxic experiment was repeated for three times.

1.5 Fluorescence imaging

Confocal fluorescent images were obtained with Zeiss LSM 800 and Olympus FV 1200 confocal laser scanning microscope. The co-localization coefficient and mean fluorescence intensity of the images were determined by the software with the Zeiss LSM 800 confocal microscope. For one-photon fluorescence imaging, ECPI-12, IVPI-12, ECPI-2, and IVPI-2, excitation = 488 nm, emission collection: 500-600 nm; MTDR and LTDR, excitation = 640 nm, emission collection: 650-750 nm. For two-photon fluorescence imaging, ECPI-12, excitation = 840 nm, emission collection: 495-540 nm; IVPI-12, excitation = 860 nm, emission collection: 495-540 nm.

2. Synthetic details, NMR spectra and HRMS spectra

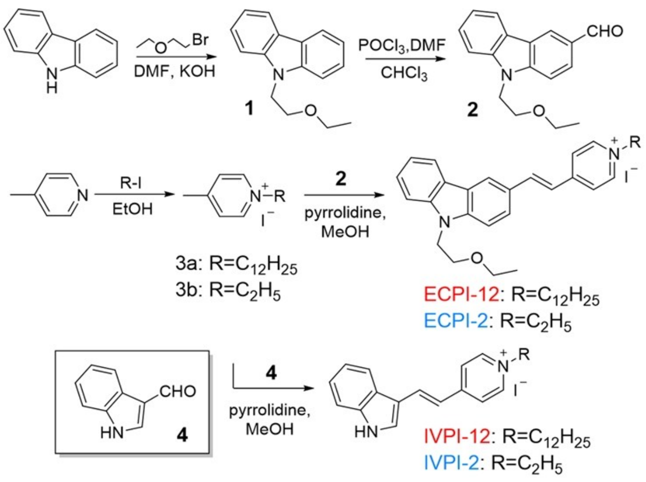

Scheme S1 Synthesis routines to ECPI-12, ECPI-2, IVPI-12, and IVPI-2.

Synthesis of compound 1: KOH (8.4 g, 150 mmol) was dissolved in *N,N*-dimethylformamide (DMF) (30 mL) and the solution was stirred at room temperature for 30 min. The DMF containing carbazole (5.0 g, 29.8 mmol) was then added and reacted for 1 h. Then 2-Bromoethyl ethyl ether (6.8 g, 45 mmol) was added dropwise into the above solution and the mixture reacted overnight. The reaction mixture was poured into water, and the yellow solid was filtrated. After recrystallization, compound **1** was obtained as a white solid (6.4 g, 90%). ^{1}H NMR (400 MHz, DMSO-d_6), δ (ppm): 8.14 (d, J = 7.60 Hz, 2H), 7.61 (d, J = 8.40 Hz, 2H), 7.44 (t, J = 7.40 Hz, 2H), 7.20 (t, J = 7.60 Hz, 2H), 4.55 (t, J = 5.40 Hz, 2H), 3.74 (t, J = 5.40 Hz, 2H), 3.33-3.39 (m, 2H), 0.97 (t, J = 7.00 Hz, 3H).

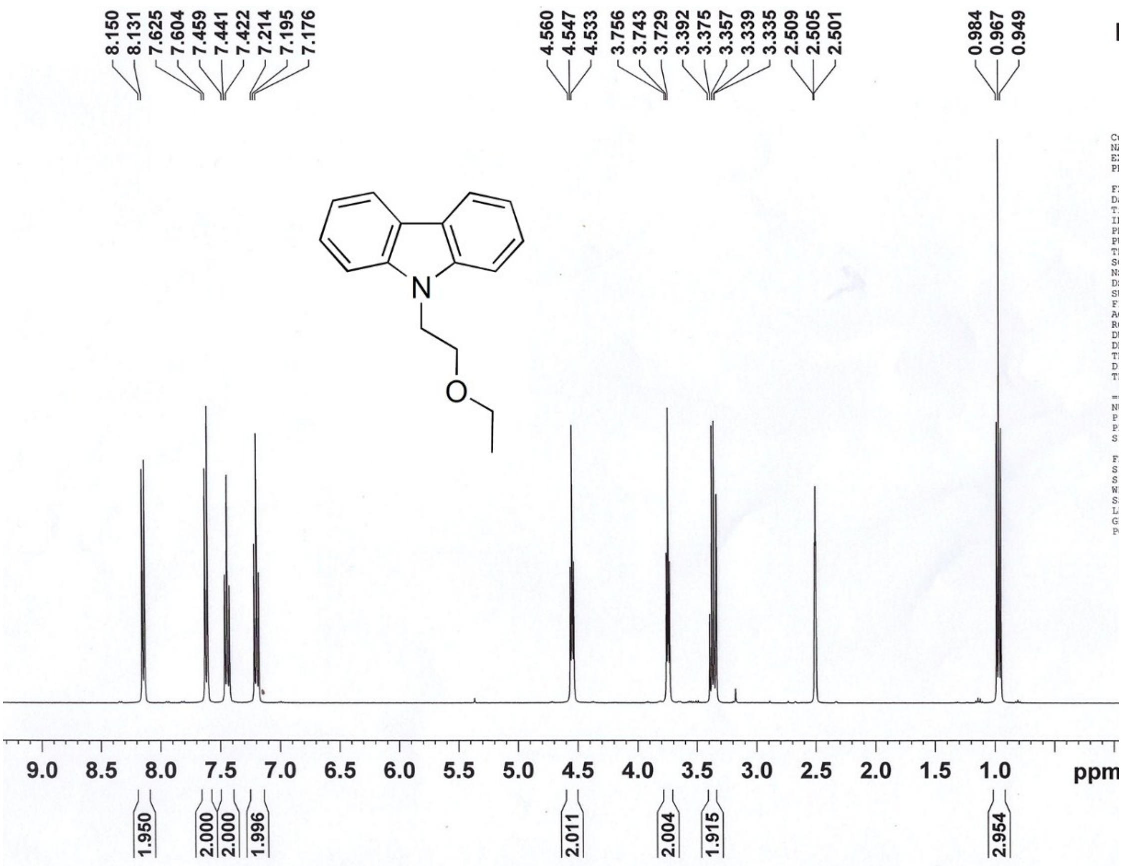

Fig. S1 ^{1}H NMR spectrum of compound **1** in DMSO-d_6

Synthesis of compound 2: Anhydrous $POCl_3$ (9.2 mL, 100 mmol) was added dropwise into anhydrous DMF (7.7 mL, 100 mmol). The mixture was cooled to 0 °C under ice bath and stirred for 30 min. Compound **1** (2.39 g, 10 mmol) dissolved in $CHCl_3$ (30 mL) was added in that mixture, and the resulting solution was stirred at room temperature for 4 h. Then the solution was stirred at 61 °C overnight. The mixture was poured into a mixture of ice and water, extracted with CH_2Cl_2. The organic layer was washed with brine and dried with anhydrous $MgSO_4$. After being dried, CH_2Cl_2 was removed under reduced pressure. Then the residue was purified by flash chromatography to give compound **2** as a white solid (1.79 g, 67%). ^{1}H NMR (300 MHz, $CDCl_3$), δ (ppm): 10.10 (s, 1H), 8.61 (d, J = 1.20 Hz, 1H), 8.15 (d, J = 7.80 Hz, 1H), 8.01 (dd, J_1= 1.50 Hz, J_2= 1.50 Hz, 1H), 7.49-7.57 (m, 3H), 7.31-7.36 (m, 1H), 4.53 (t, J = 5.85 Hz, 2H), 3.83 (t, J = 5.85 Hz, 2H), 3.41 (q, J = 7.00 Hz, 2H), 1.09 (t, J = 7.05 Hz, 3H).

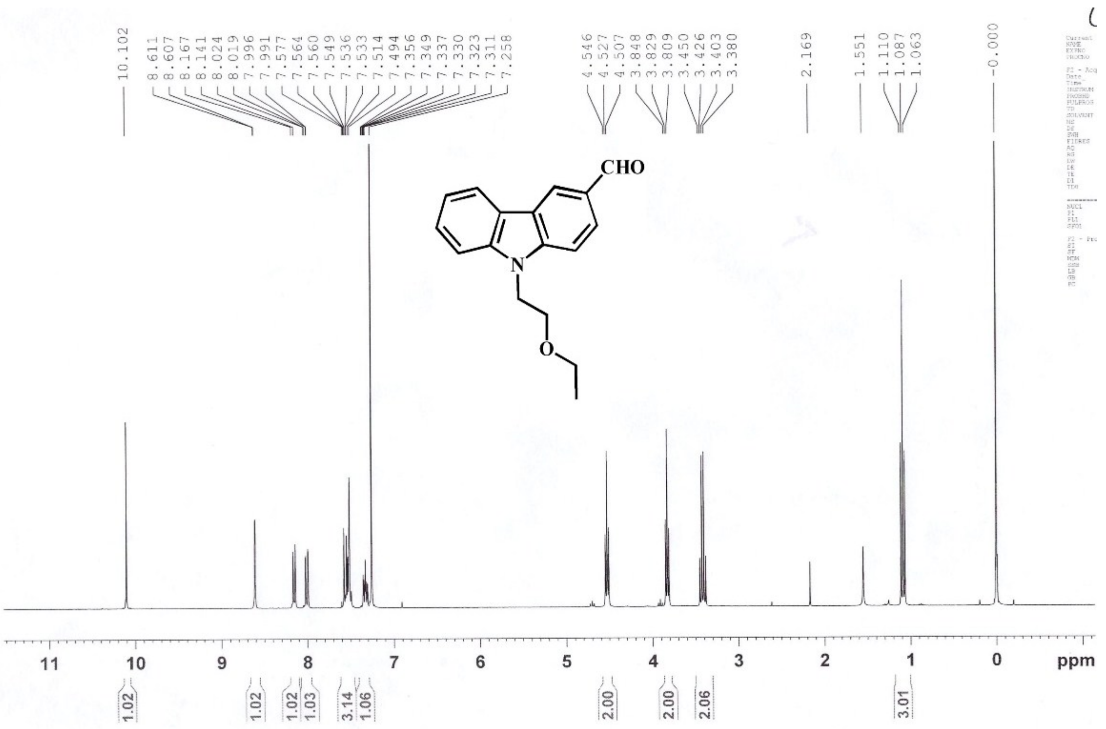

Fig. S2 ^{1}H NMR spectrum of compound **2** in $CDCl_3$

Synthesis of Compound 3a: 4-methylpyridine (1mL, 10 mmol) was dissolved in ethanol (5 mL) and 1-iodododecane (3 mL, 12 mmol) was added. The mixture was stirred at 78 °C overnight. Then the reaction mixture was poured into petroleum ether, and the yellow solid was filtrated. After recrystallization, compound **3a** was obtained as a yellow solid (3.54 g, 91%). ^{1}H NMR (400 MHz, DMSO-d_6), δ (ppm): 8.93 (d, J = 6.40 Hz, 2H), 7.99 (d, J = 6.40 Hz, 2H), 4.51 (t, J = 7.40 Hz, 2H), 2.61 (s, 3H), 1.89 (q, J = 7.20 Hz, 2H), 1.23 (s, 18H), 0.85 (t, J = 6.80 Hz, 3H).

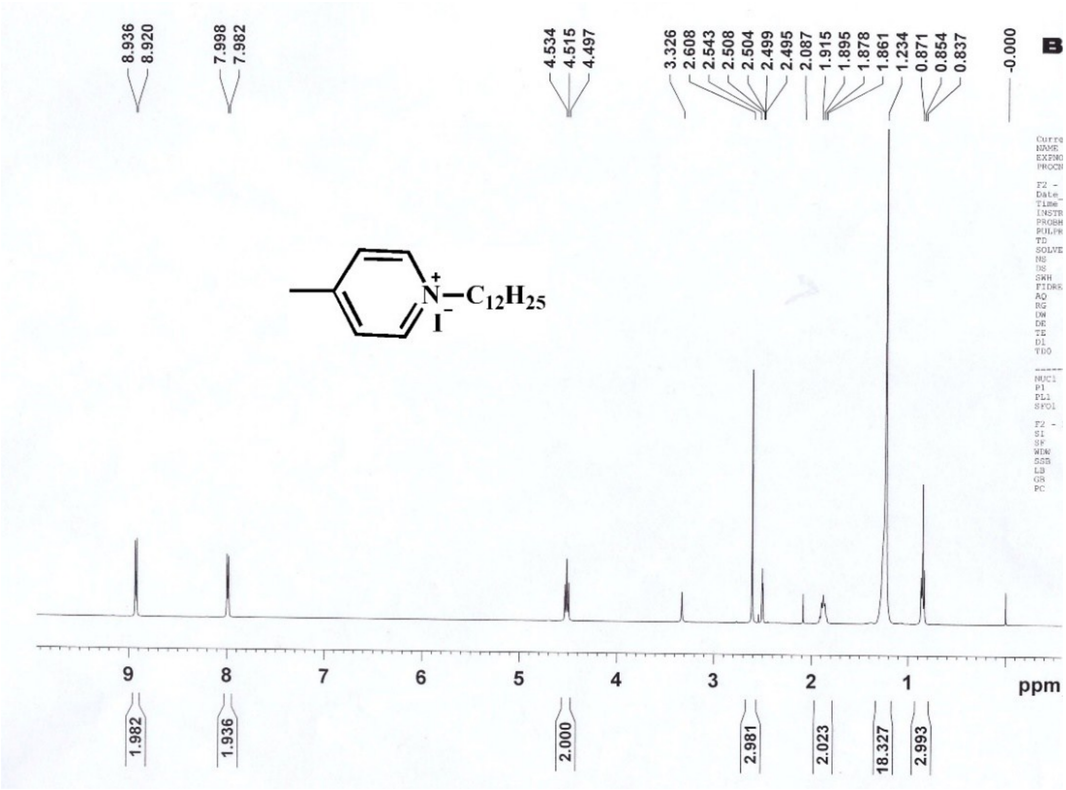

Fig. S3 ^{1}H NMR spectrum of compound **3a** in DMSO-d_6

Synthesis of Compound 3b: 4-methylpyridine (1mL, 10 mmol) was dissolved in ethanol (5 mL) and iodoethane (1 mL, 12 mmol) was added. The mixture was stirred at 78 °C overnight. Then the reaction mixture was poured into petroleum ether, and the light yellow solid was filtrated. After recrystallization, compound **3b** was obtained as a light yellow solid (2.24 g, 90%). ^{1}H NMR (300 MHz, D_2O), δ (ppm): 8.55 (d, J = 6.30 Hz, 2H), 7.77 (d, J = 6.00 Hz, 2H), 4.46 (q, J = 7.40 Hz, 2H), 2.55 (s, 3H), 1.51 (q, J = 7.35 Hz, 3H).

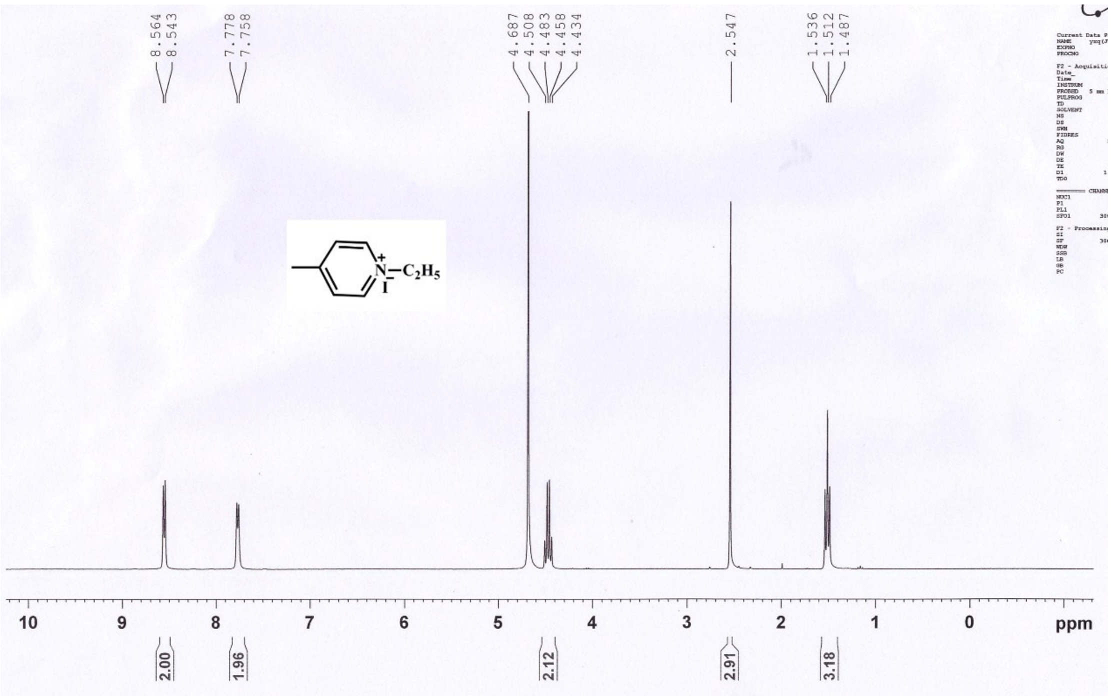

Fig. S4 ^{1}H NMR spectrum of compound **3b** in D_2O

Synthesis of ECPI-12: Pyrrolidine (200 μL) was added to the solution of compound **2** (0.27 g, 1 mmol) and compound **3a** (0.39 g, 1 mmol) dissolved in methanol (5 mL). The mixture was stirred at room temperature for 5 h. When the reaction mixture was poured into petroleum ether, the yellow solid was filtrated. After recrystallization, ECPI-12 was obtained as a yellow solid (0.43 g, 67 %). ^{1}H NMR (300 MHz, DMSO-d_6), δ (ppm): 8.90 (d, J = 6.90 Hz, 2H), 8.56 (s, 1H), 8.21 (m, 4H), 7.89 (dd, J_1 = 8.70 Hz, J_2 = 1.20 Hz, 1H), 7.75 (d, J = 8.70 Hz, 1H), 7.69 (d, J = 8.10 Hz, 1H), 7.49-7.56 (m, 2H), 7.29 (t, J = 7.35 Hz, 1H), 4.61 (t, J = 5.25 Hz, 2H), 4.47 (t, J = 7.20 Hz, 2H), 3.77 (t, J = 5.10 Hz, 2H), 3.32-3.41 (m, 2H), 1.91 (m, 2H), 1.26 (m, 18H), 0.96 (t, J = 7.05 Hz, 3H), 0.84 (t, J = 6.60 Hz, 3H). ^{13}C NMR (400 MHz, DMSO-d_6), δ (ppm): 153.98, 144.39, 143.11, 142.35, 141.37, 126.82, 126.76, 126.54, 123.57, 123.17, 122.54, 121.54, 120.75, 120.46, 120.24, 110.96, 110.69, 68.63, 66.11, 59.97, 43.41, 31.74, 30.93, 29.45, 29.34, 29.22, 29.15, 28.83, 25.89, 22.53, 15.43, 14.39. HRMS m/z: calcd for $C_{35}H_{47}N_2O^+$ 511.3683 ([M-I]$^+$); found 511.3600.

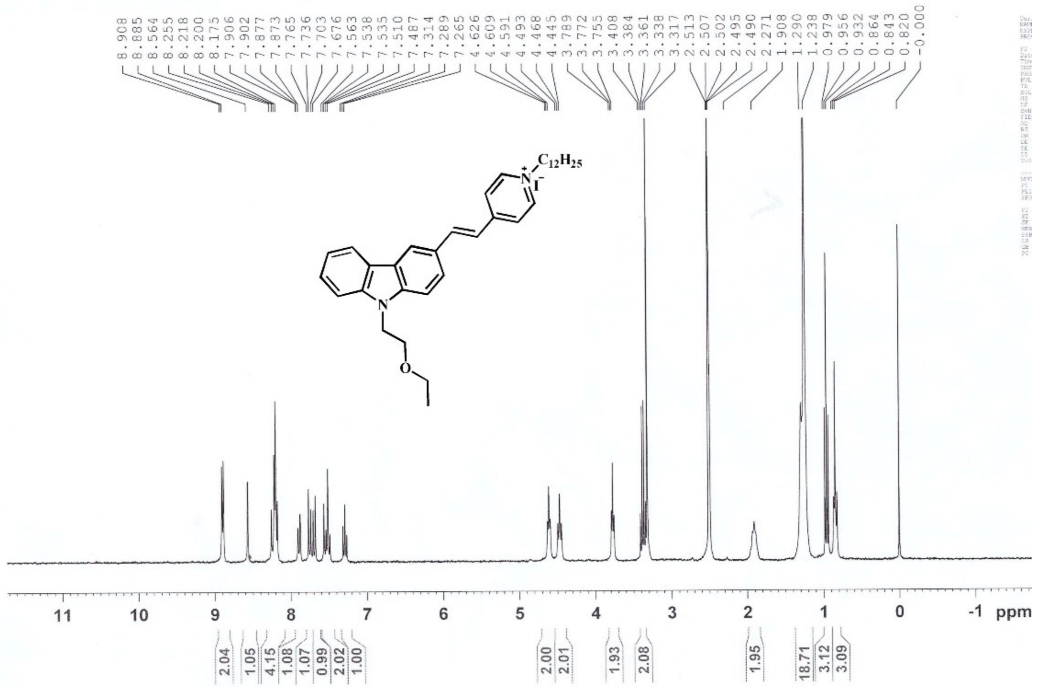

Fig. S5 ^{1}H NMR spectrum of ECPI-12 in DMSO-d_6

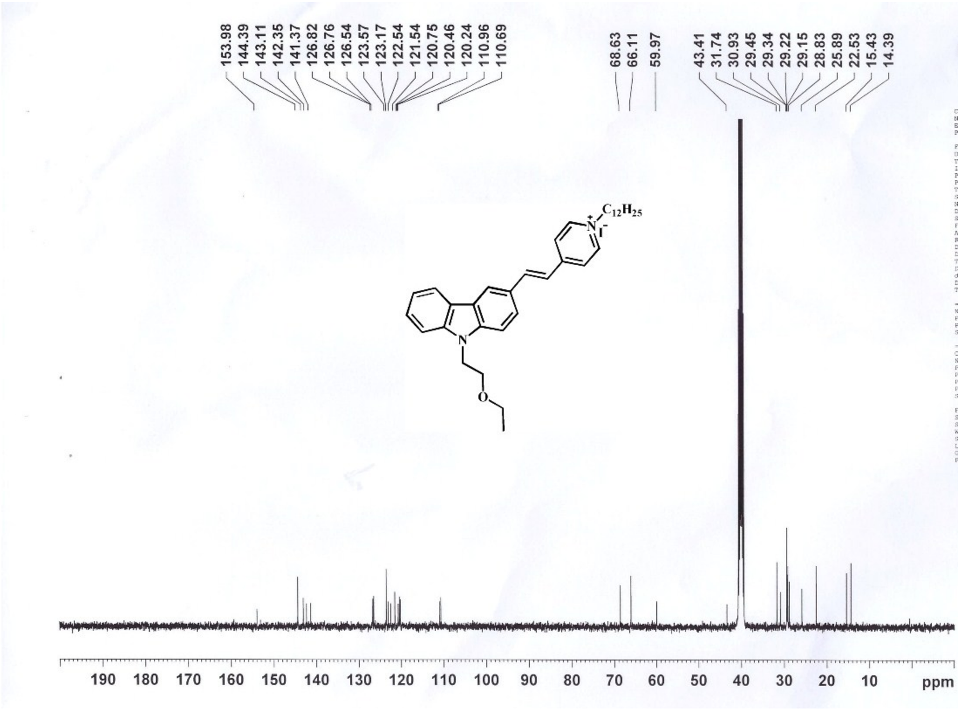

Fig. S6 ^{13}C NMR spectrum of ECPI-12 in DMSO-d_6

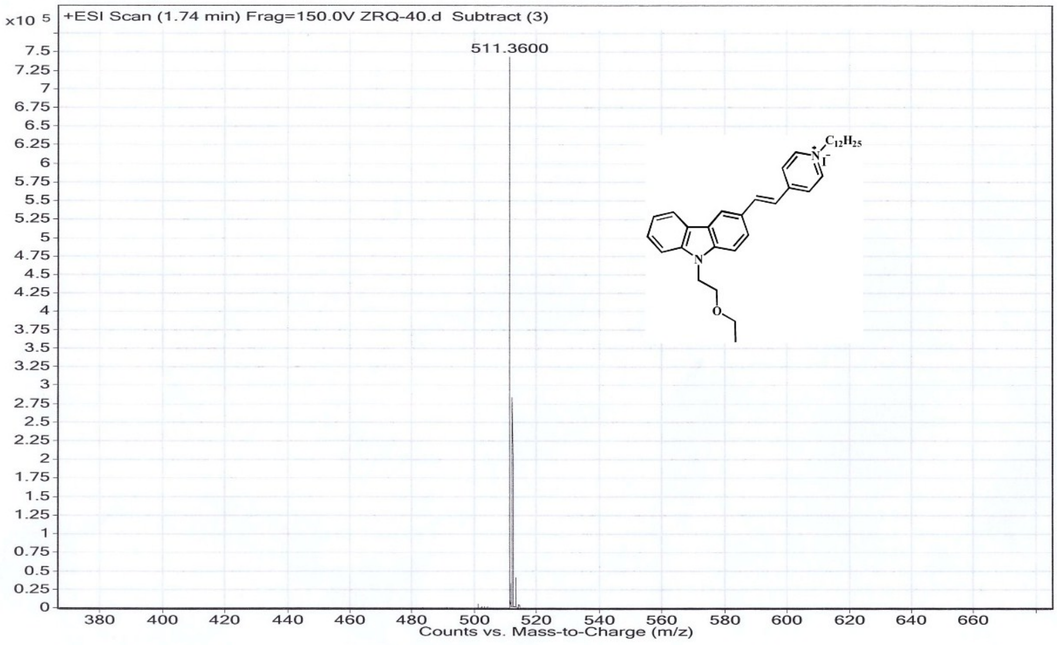

Fig. S7 HRMS of ECPI-12

Synthesis of ECPI-2: The experimental procedure was similar to the synthetic process of ECPI-12. ECPI-2 was obtained as a yellow solid (0.35 g, 70%). ^{1}H NMR (400 MHz, $CDCl_3$) δ (ppm): 8.87 (d, J = 5.20 Hz, 2H), 8.31 (s, 1H), 8.12 (d, J = 7.60 Hz, 1H), 7.98 (d, J = 5.20 Hz, 2H), 7.86 (d, J = 16.00 Hz, 1H), 7.74 (d, J = 8.40 Hz, 1H), 7.43-7.52 (m, 3H), 7.26-7.32 (m, 1H), 7.14 (d, J = 16.00 Hz, 1H), 4.65 (d, J = 7.20 Hz, 2H), 4.43 (t, J = 5.60 Hz, 2H), 3.80 (t, J = 5.60 Hz, 2H), 3.42 (q, J = 6.93 Hz, 2H), 1.63 (q, J = 7.20 Hz, 3H), 1.10 (d, J = 7.20 Hz, 3H). ^{13}C NMR (400 MHz, DMSO-d_6), δ (ppm): 153.90, 144.19, 142.97, 142.31, 141.35, 126.82, 126.75, 126.54, 123.59, 123.14, 122.53, 121.53, 120.77, 120.46, 120.23, 110.96, 110.69, 68.63, 66.10, 55.46, 43.38, 16.63, 15.44. HRMS m/z: calcd for $C_{25}H_{27}N_2O^+$ 371.2118 ([M-I]$^+$); found 371.2107.

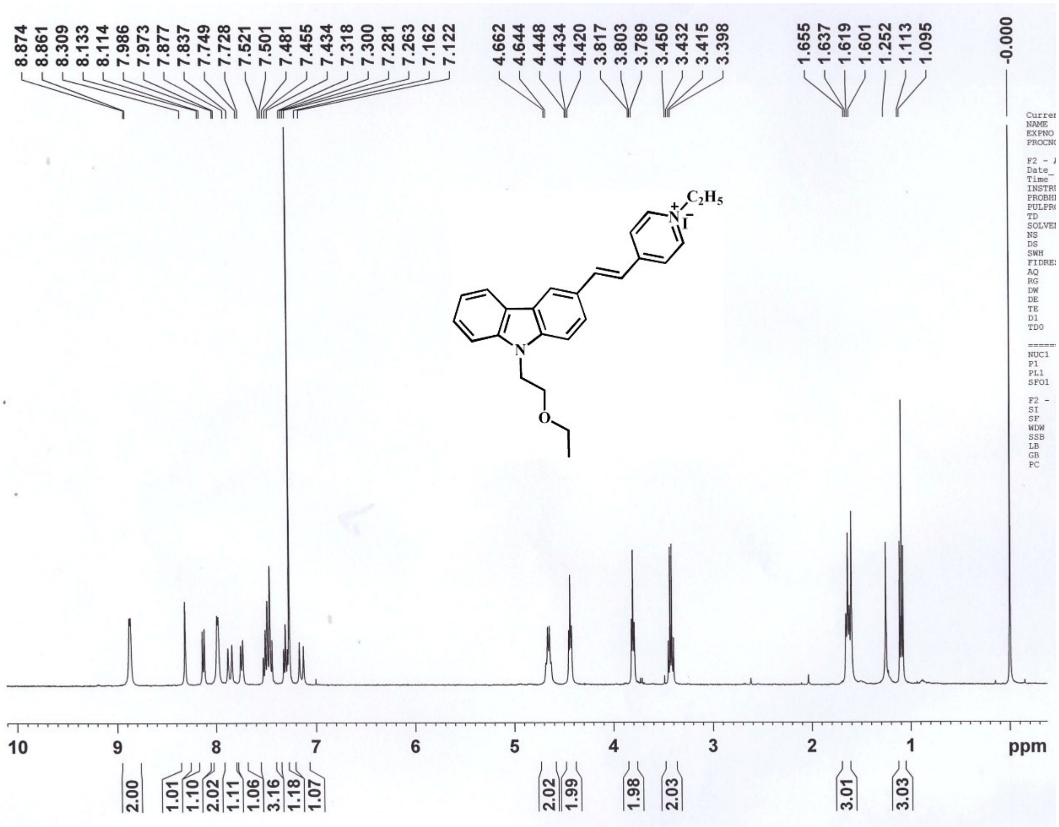

Fig. S8 ^{1}H NMR spectrum of ECPI-2 in $CDCl_3$

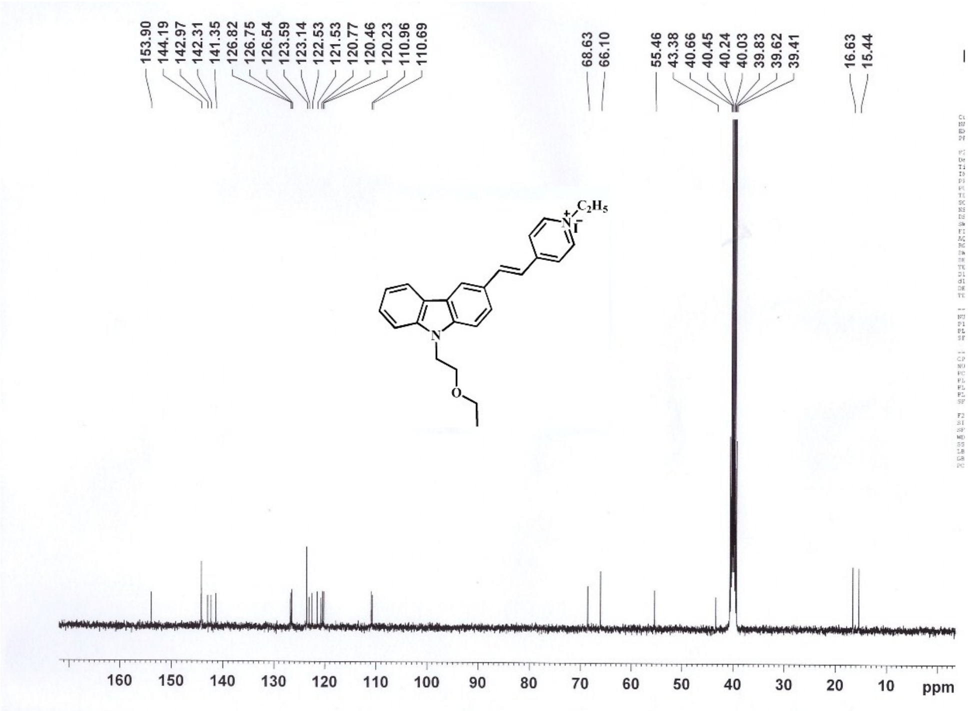

Fig. S9 ^{13}C NMR spectrum of ECPI-2 in DMSO-d_6

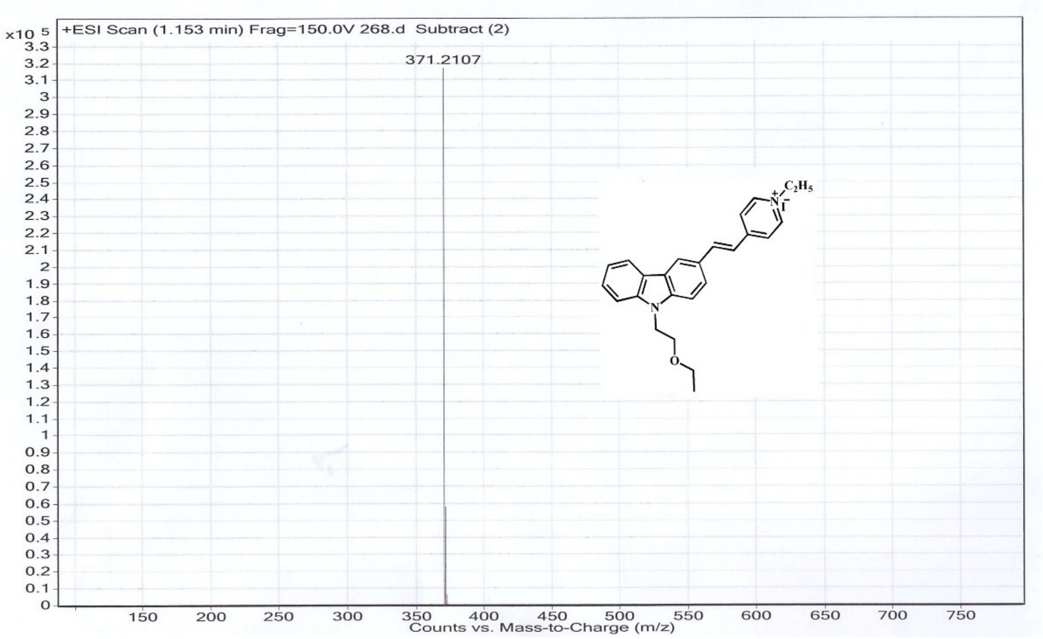

Fig. S10 HRMS of ECPI-2

Synthesis of IVPI-12: Pyrrolidine (200 µL) was added to the solution of compound **4** (0.15 g, 1 mmol) and compound **3a** (0.39 g, 1 mmol) dissolved in methanol (5 mL). The mixture was stirred at room temperature for 5 h. Then the reaction mixture was poured into petroleum ether, and the orange red solid was filtrated. After recrystallization, IVPI-12 was obtained as an orange red solid (0.38 g, 73 %). ^{1}H NMR (300 MHz, $CDCl_3$), δ (ppm): 11.14 (s, 1H), 7.76-7.63 (m, 5H), 7.62 (d, J = 3.60 Hz, 1H), 7.33 (s, 1H), 7.27 (s, 1H), 7.19 (d, J = 2.40 Hz, 2H), 6.58 (d, J = 15.60 Hz, 1H), 4.25 (s, 1H), 1.75 (m, 2H), 1.23 (m, 18H), 0.87 (t, J = 6.60 Hz, 3H). ^{13}C NMR (400 MHz, DMSO-d_6), δ (ppm): 155.03, 143.78, 138.03, 137.02, 132.85, 125.40, 123.39, 122.36, 121.61, 120.90, 117.28, 114.12, 113.07, 59.51, 31.74, 30.89, 29.45, 29.34, 29.23, 29.15, 28.84, 25.89, 22.53, 14.38. HRMS m/z: calcd for $C_{27}H_{37}N_2^+$ 389.2951 ([M-I]$^+$); found 389.2573.

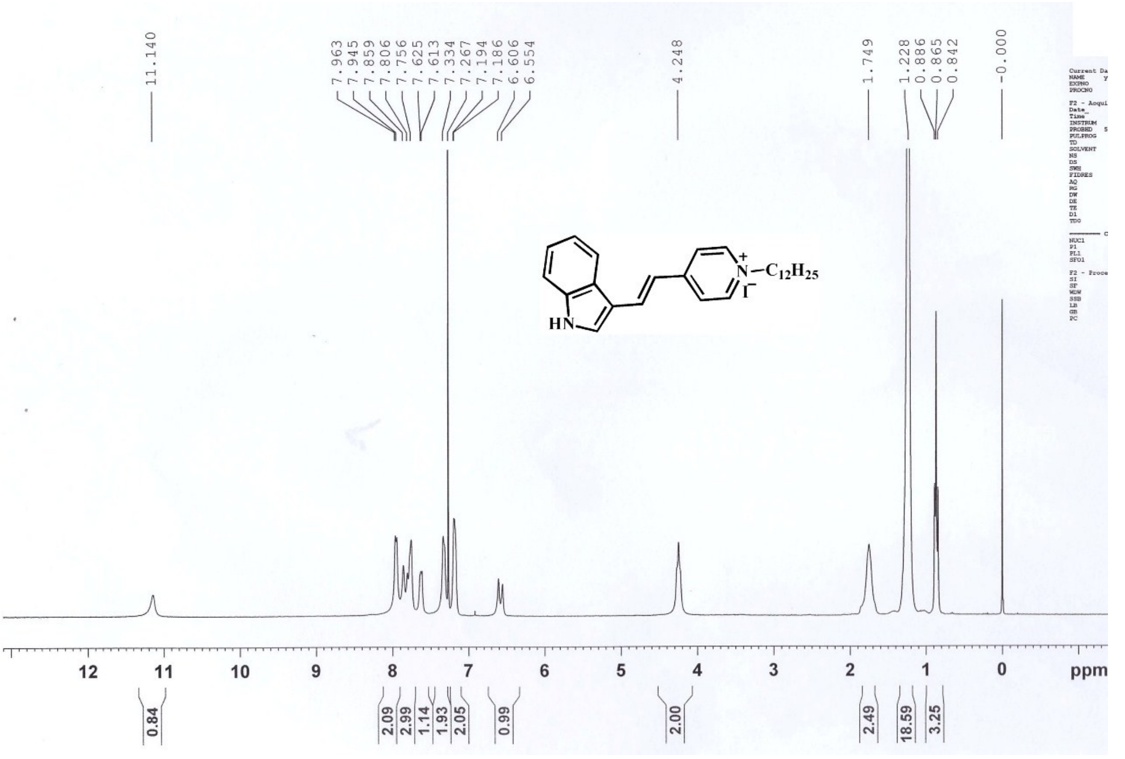

Fig. S11 ^{1}H NMR spectrum of IVPI-12 in $CDCl_3$

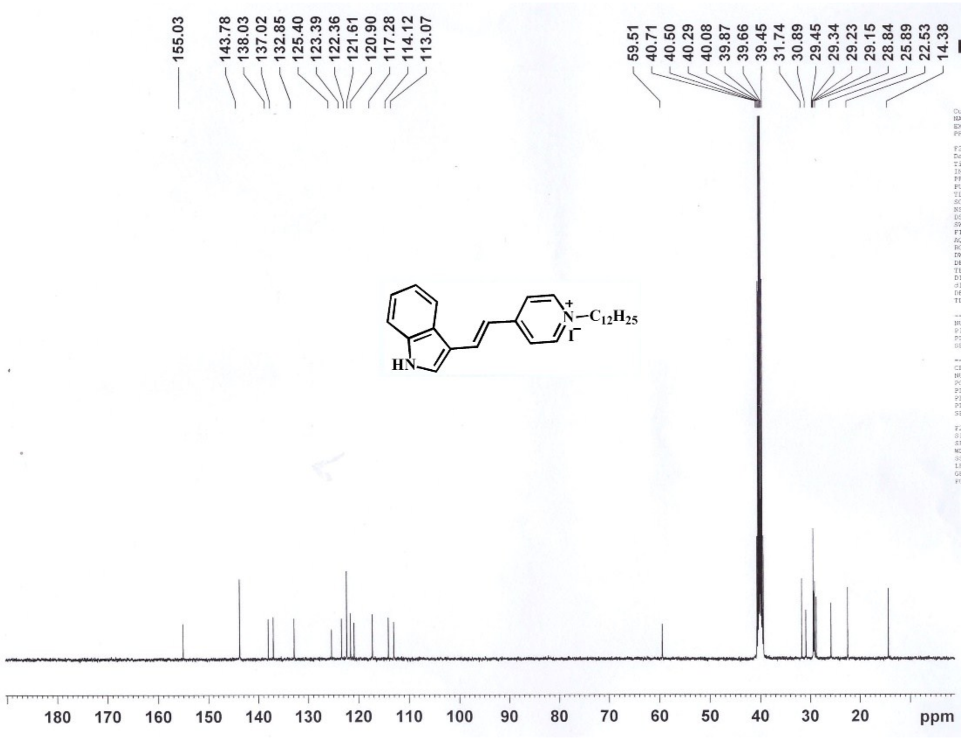

Fig. S12 ^{13}C NMR spectrum of IVPI-12 in DMSO-d_6

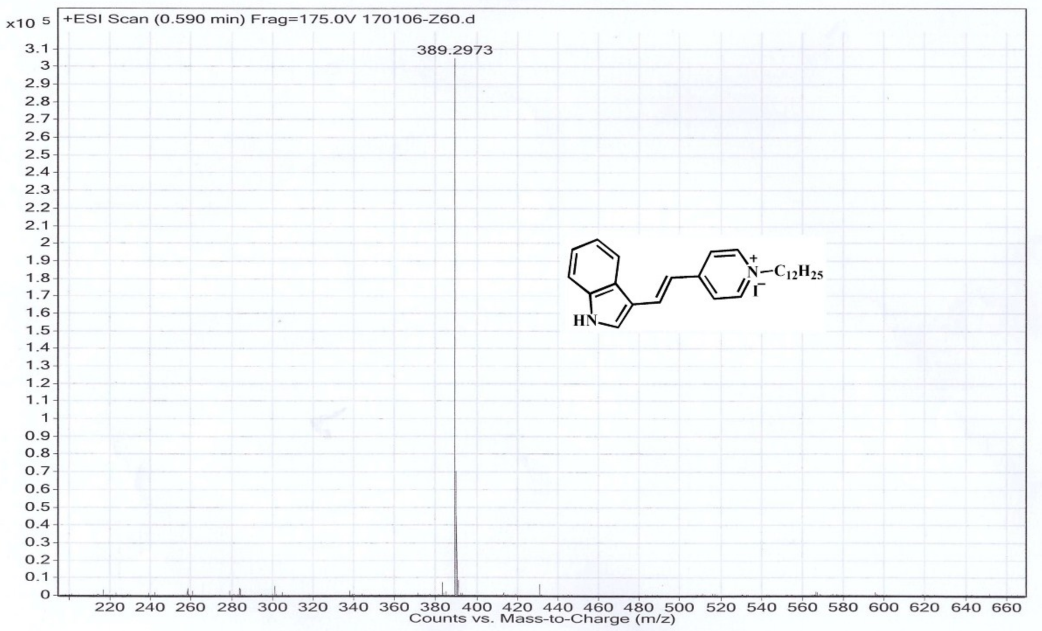

Fig. S13 HRMS of IVPI-12

Synthesis of IVPI-2: The experimental procedure was similar to that of IVPI-12. IVPI-2 was obtained as a red solid (0.26 g, 68%). ^{1}H NMR (300 MHz, DMSO-d_6) δ (ppm): 11.93 (s, 1H), 8.79 (d, J = 6.90 Hz, 2H), 8.26 (d, J = 16.20 Hz, 1H), 8.13-8.18 (m, 3H), 7.97 (d, J = 2.70 Hz, 1H), 7.52 (dd, J_1 = 6.30 Hz, J_2 = 2.40 Hz, 1H), 7.21-7.33 (m, 3H), 4.45 (q, J = 7.30 Hz, 2H), 1.52 (t, J = 7.35 Hz, 3H). ^{13}C NMR (400 MHz, DMSO-d_6), δ (ppm): 154.97, 143.58, 138.00, 136.89, 132.82, 125.36, 123.40, 122.40, 121.61, 120.92, 117.29, 114.07, 113.07, 54.99, 16.62. HRMS m/z: calcd for $C_{17}H_{17}N_2^+$ 249.1386 ([M-I]$^+$); found 249.1396.

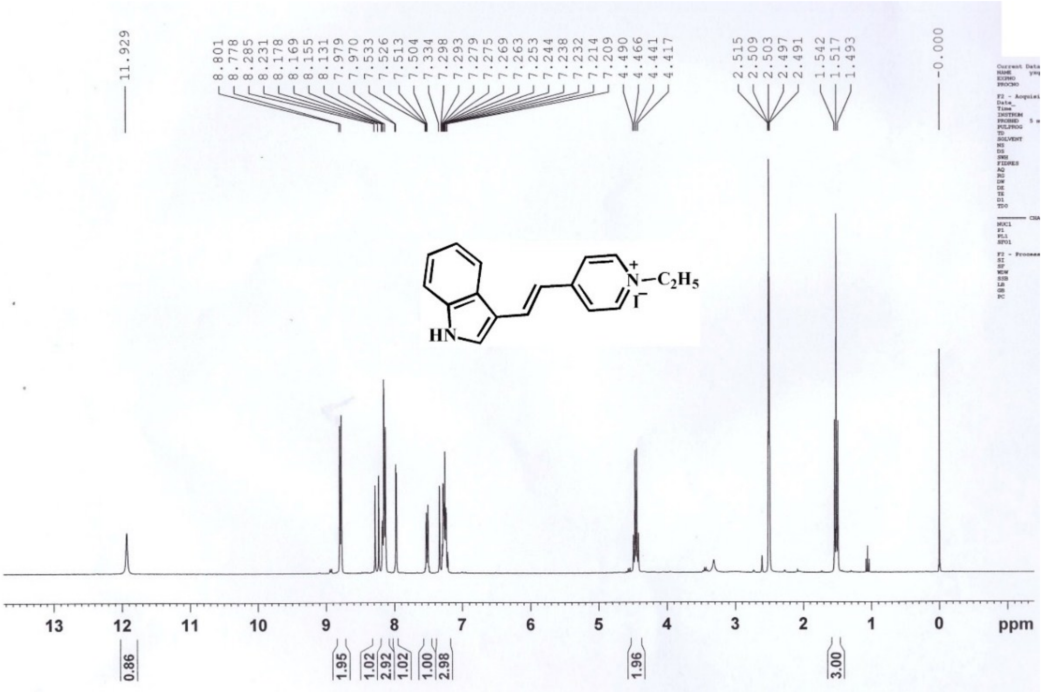

Fig. S14 ^{1}H NMR spectrum of IVPI-2 in DMSO-d_6

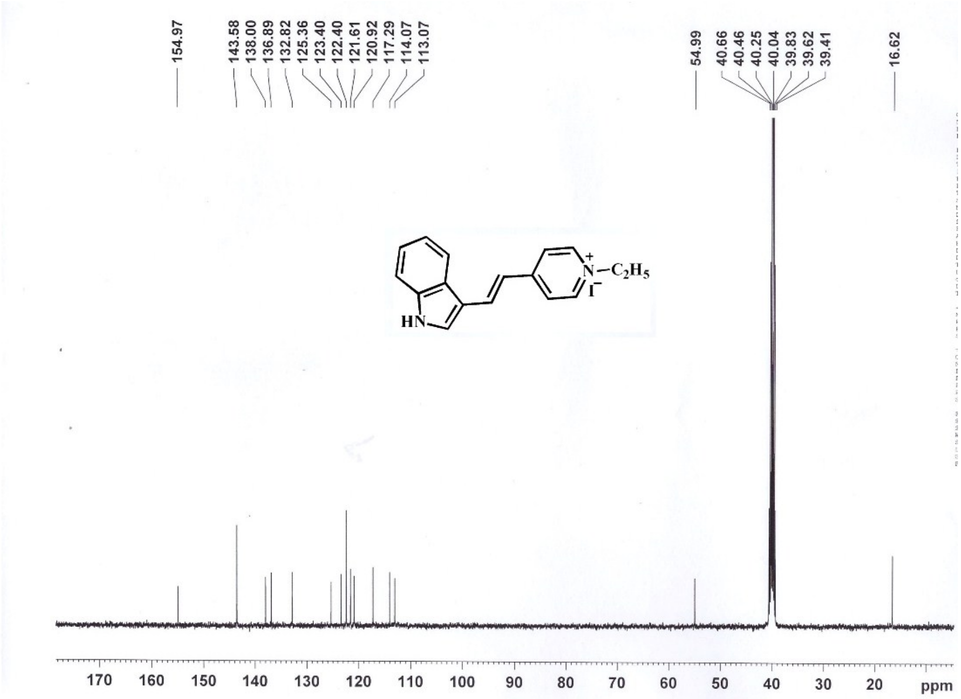

Fig. S15 ^{13}C NMR spectrum of IVPI-2 in DMSO-d_6

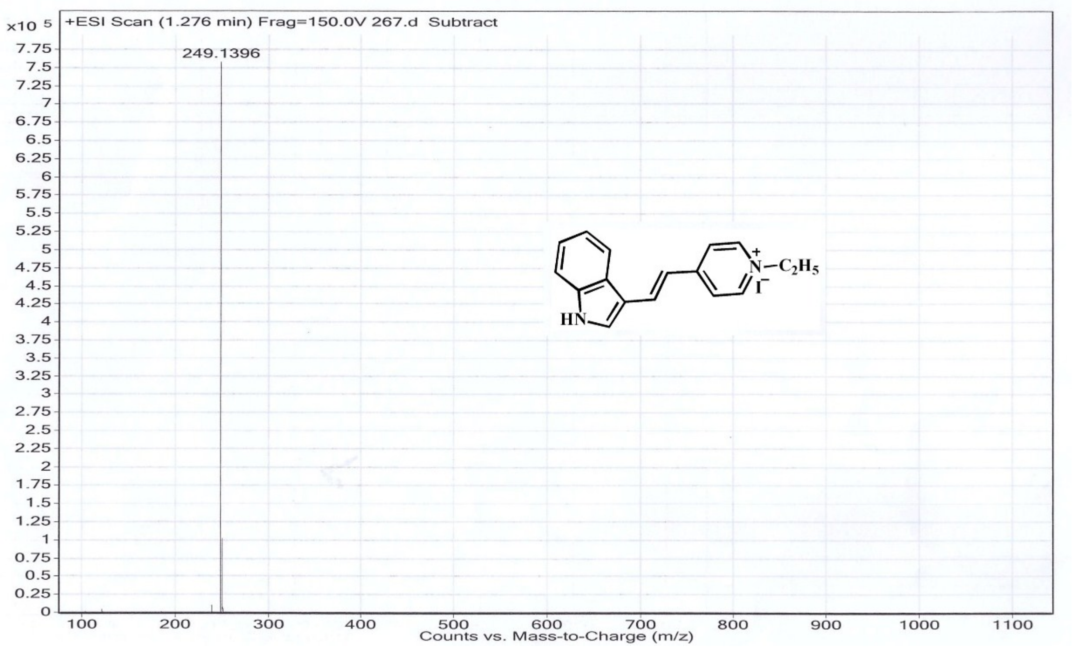

Fig. S16 HRMS of IVPI-2

3. Crystallographic data

Table S1 Crystal data and structure refinement for ECPI-12.

Bond precision:	C-C = 0.0184 A	Wavelength=1.54184	
Cell:	a=6.9216(2)	b=59.8833(15)	c=7.8371(4)
	alpha=90	beta=94.192(3)	gamma=90
Temperature:	173 K		

	Calculated	Reported
Volume	3239.7(2)	3239.7(2)
Space group	P 21/c	P 1 21/c 1
Hall group	-P 2ybc	-P 2ybc
Moiety formula	C35 H47 N2 O, I	I, C35 H47 N2 O
Sum formula	C35 H47 I N2 O	C35 H47 I N2 O
Mr	638.65	638.64
Dx,g cm-3	1.309	1.309
Z	4	4
Mu (mm-1)	7.967	7.967
F000	1328.0	1328.0
F000'	1329.83	
h,k,lmax	8,69,9	8,69,9
Nref	5318	5236
Tmin,Tmax	0.705,0.853	0.055,1.000
Tmin'	0.639	

Correction method= # Reported T Limits: Tmin=0.055 Tmax=1.000
AbsCorr = MULTI-SCAN

Data completeness= 0.985 Theta(max)= 63.684

R(reflections)= 0.1135(4443) wR2(reflections)= 0.2764(5236)

S = 1.129 Npar= 354

Table S2 Crystal data and structure refinement for IVPI-12.

Bond precision:	C-C = 0.0081 A	Wavelength=1.54184	
Cell:	a=10.5322(2)	b=16.7869(4)	c=43.8778(8)
	alpha=90	beta=90.202(2)	gamma=90
Temperature:	173 K		

	Calculated	Reported
Volume	7757.7(3)	7757.7(3)
Space group	P 21/n	P 1 21/n 1
Hall group	-P 2yn	-P 2yn
Moiety formula	C27 H37 N2, I	I, C27 H37 N2
Sum formula	C27 H37 I N2	C27 H37 I N2
Mr	516.49	516.48
Dx,g cm-3	1.327	1.327
Z	12	12
Mu (mm-1)	9.822	9.822
F000	3192.0	3192.0
F000’	3195.44	
h,k,lmax	12,20,52	12,20,52
Nref	13856	12995
Tmin,Tmax	0.448,0.456	0.300,1.000
Tmin’	0.326	

Correction method= # Reported T Limits: Tmin=0.300 Tmax=1.000
AbsCorr = MULTI-SCAN

Data completeness= 0.938 Theta(max)= 67.080

R(reflections)= 0.0434(11269) wR2(reflections)= 0.1182(12995)

S = 0.905 Npar= 814

4. Photophysical data

Table S3 One-photon photophysical properties of ECPI-12, IVPI-12, ECPI-2, and IVPI-2

Probe	Solvent	λ_{abs}^{max} (nm)	λ_{em}^{max} (nm)	ε ($M^{-1}cm^{-1}$)	Φ (%)
ECPI-12	DMSO	432	561	3.20×10^4	10.89
	Water	412	558	2.10×10^4	1.23
IVPI-12	DMSO	436	529	4.14×10^4	3.39
	Water	420	527	2.16×10^4	0.85
ECPI-2	DMSO	428	560	2.70×10^4	11.70
	Water	416	553	3.08×10^4	1.28
IVPI-2	DMSO	434	532	2.16×10^4	2.97
	Water	418	529	2.34×10^4	0.42

λ_{abs}^{max} and λ_{em}^{max} are the maximum absorption and one-photon fluorescence wavelengths, respectively. ε is molar extinction coefficient. Φ is one-photon fluorescence quantum yield determined by using fluorescein (Φ = 0.95) as the standard. Concentration: 10 μM.

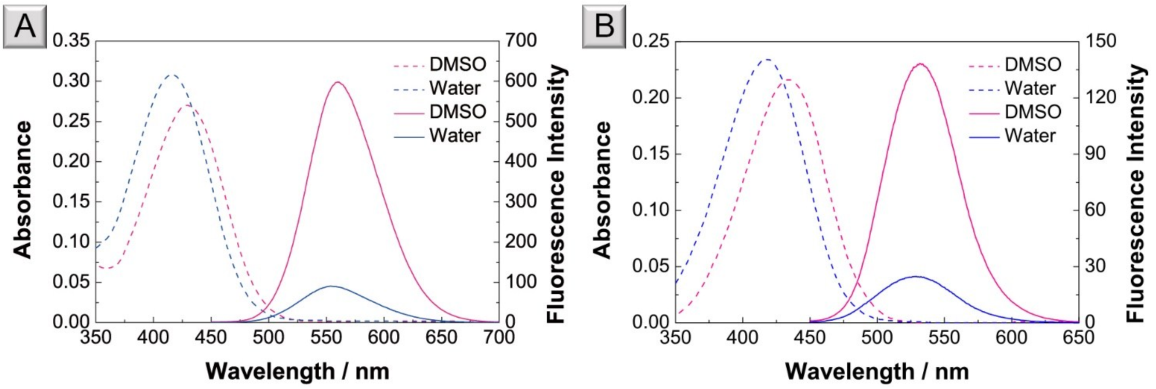

Fig. S17 Absorption (dashed line) and fluorescence spectra (solid line) of ECPI-2 (A) and IVPI-2 (B) in DMSO and water. Concentration: 10 μM.

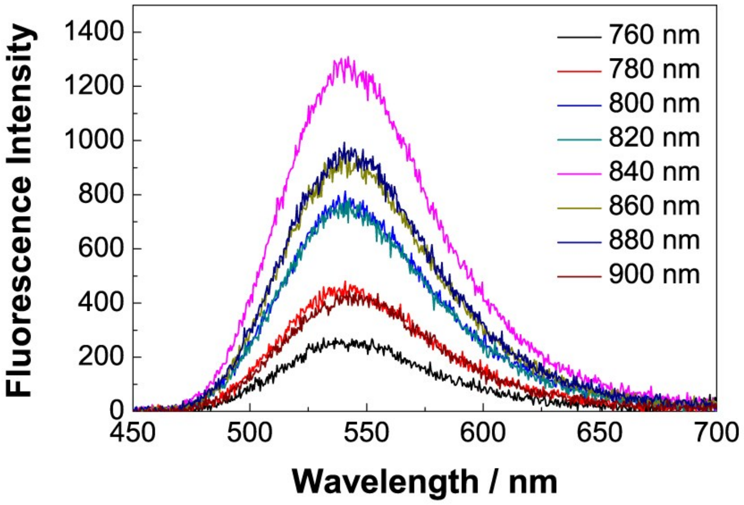

Fig. S18 TPEF spectra of IVPI-12 in DMSO excited by 760, 780, 800, 820, 840, 860, 880, and 900 nm, respectively. Concentration: 10 μM.

Table S4 δ value of ECPI-12 and IVPI-12

λ / nm	760	780	800	820	840	860	880	900
ECPI-12	659	853	880	1085	1412	1402	727	569
IVPI-12	24	28	26	39	50	58	32	30

δ (GM): two-photon absorption cross-sections. Concentration: 10 μM.

5. Imaging data

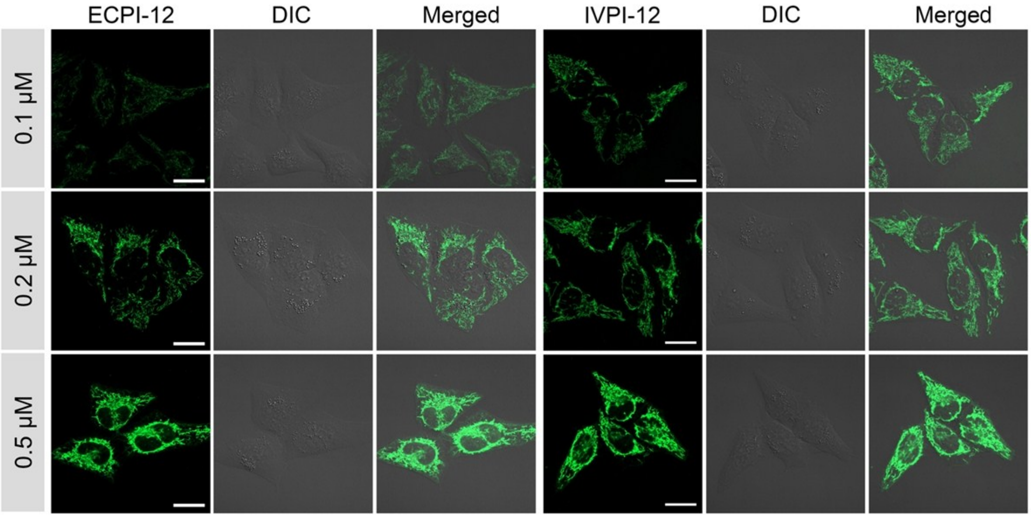

Fig. S19 CLSM images of live HeLa cells stained with ECPI-12 and IVPI-12 at different concentrations for 30 min. Scale bar = 20 μm.

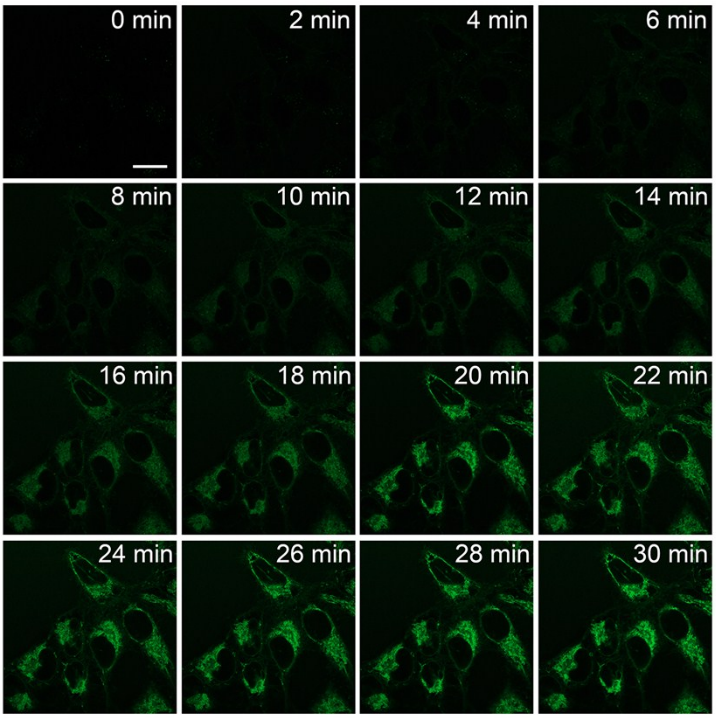

Fig. S20 CLSM images of live HeLa cells stained with 0.2 μM ECPI-12 at different time points. Scale bar = 20 μm.

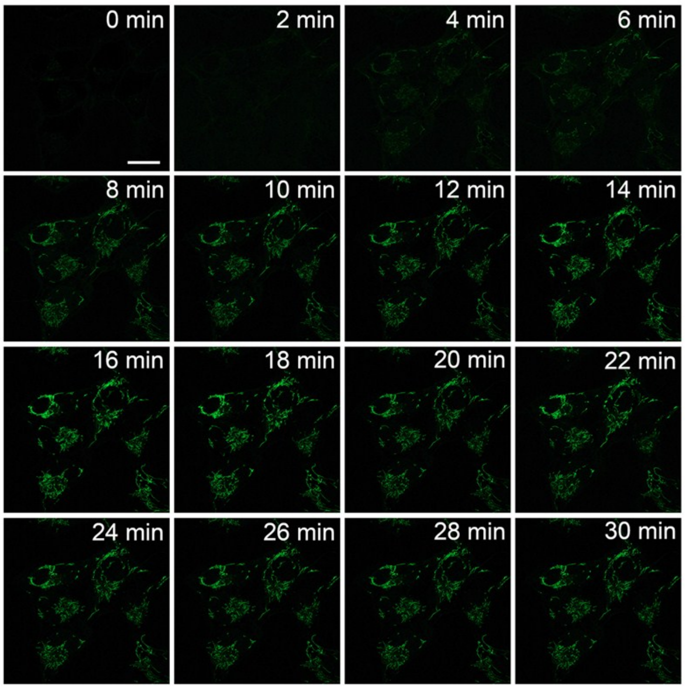

Fig. S21 CLSM images of live HeLa cells stained with 0.2 μM IVPI-12 at different time points. Scale bar = 20 μm.

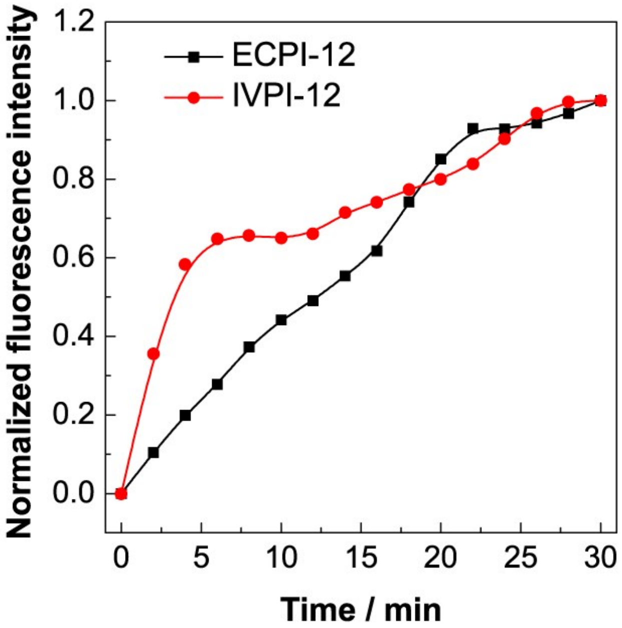

Fig. S22 Normalized mean fluorescence intensity of 0.2 μM ECPI-12 and 0.2 μM IVPI-12 in live HeLa cells at different time points.

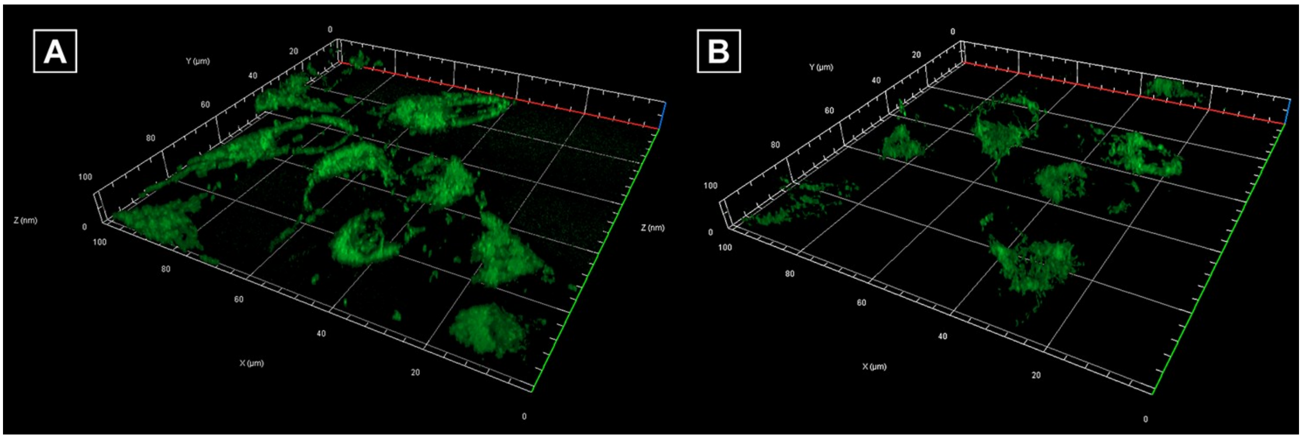

Fig. S23 Reconstructed 3D fluorescent images of live HeLa cells stained with 0.2 μM ECPI-12 (A) and 0.2 μM IVPI-12 (B) at different depths along the *Z* axis for 30 min.

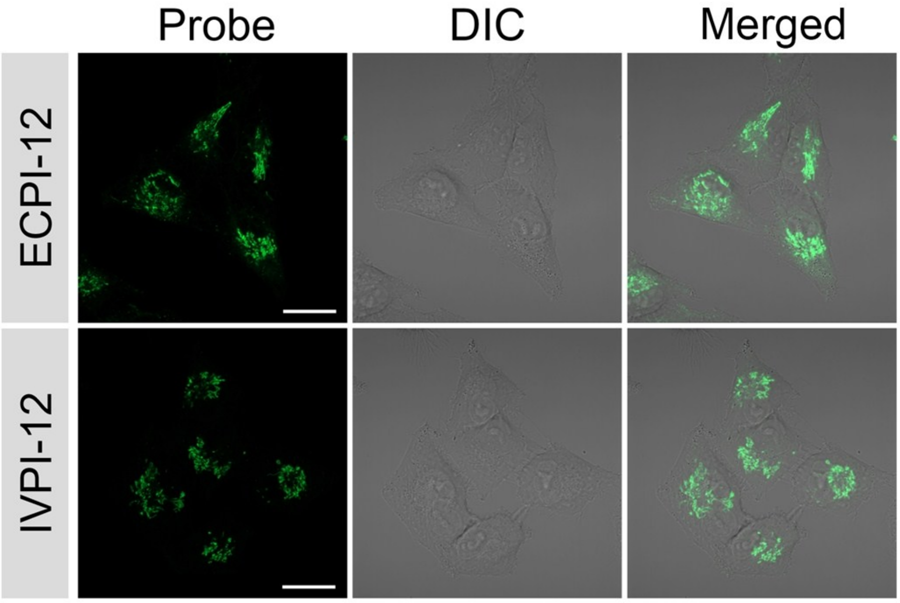

Fig. S24 CLSM images of live A549 cells stained with 0.2 μM ECPI-12 and 0.2 μM IVPI-12 for 30 min. Scale bar = 20 μm.

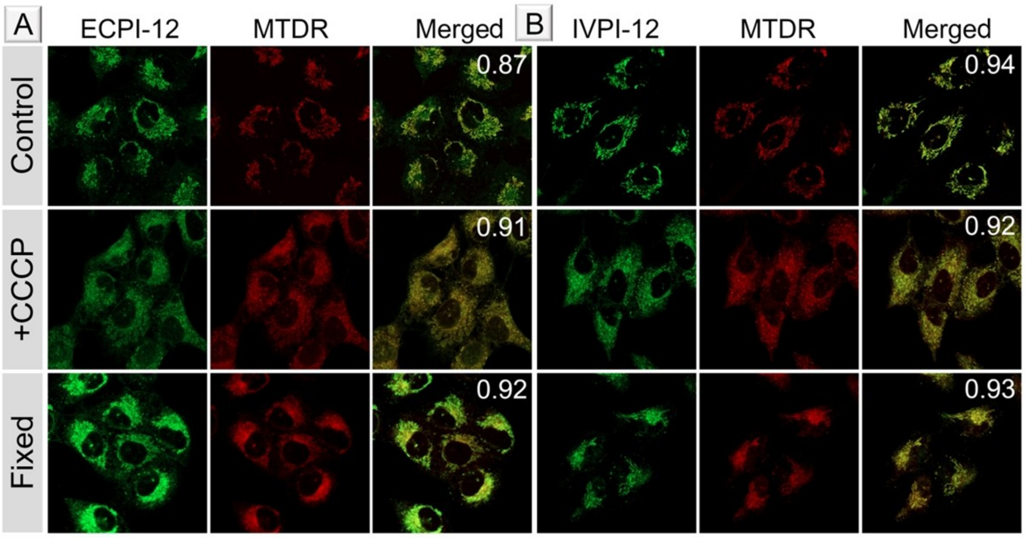

Fig. S25 CLSM images of live, CCCP-treated and fixed A549 cells stained with 0.2 μM ECPI-12 (A) or IVPI-12 (B) and 0.2 μM MTDR. Co-localization coefficients of ECPI-12 or IVPI-12 and MTDR were shown in the merged images. Scale bar = 20 μm.

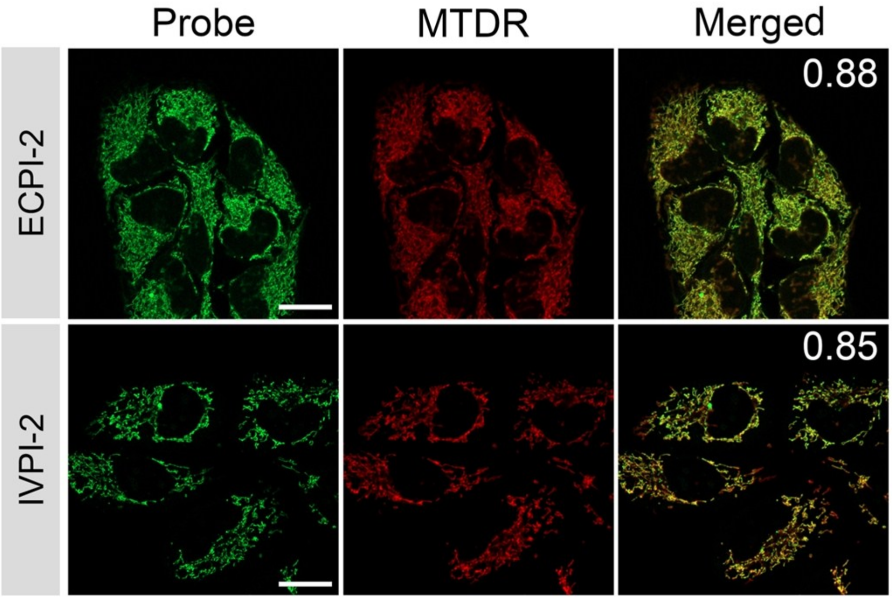

Fig. S26 Co-stain images of live HeLa cells stained with 0.2 μM ECPI-2 or IVPI-2 and 0.2 μM MTDR for 30 min. Co-localization coefficient of ECPI-2 or IVPI-2 and MTDR was shown in the merged images. Scale bar = 20 μm.

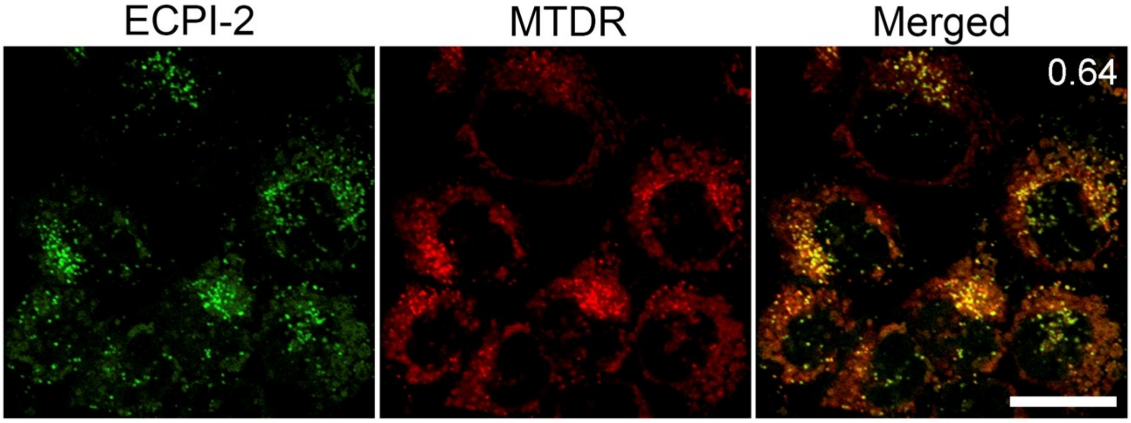

Fig. S27 Co-stain images of live HeLa cells stained with ECPI-2 (0.2 μM, 30 min) and MTDR (0.2 μM, 1 h), and then treated with 15 μM CCCP for 20 min. Co-localization coefficient of ECPI-2 and MTDR was shown in the merged image. Scale bar = 20 μm.

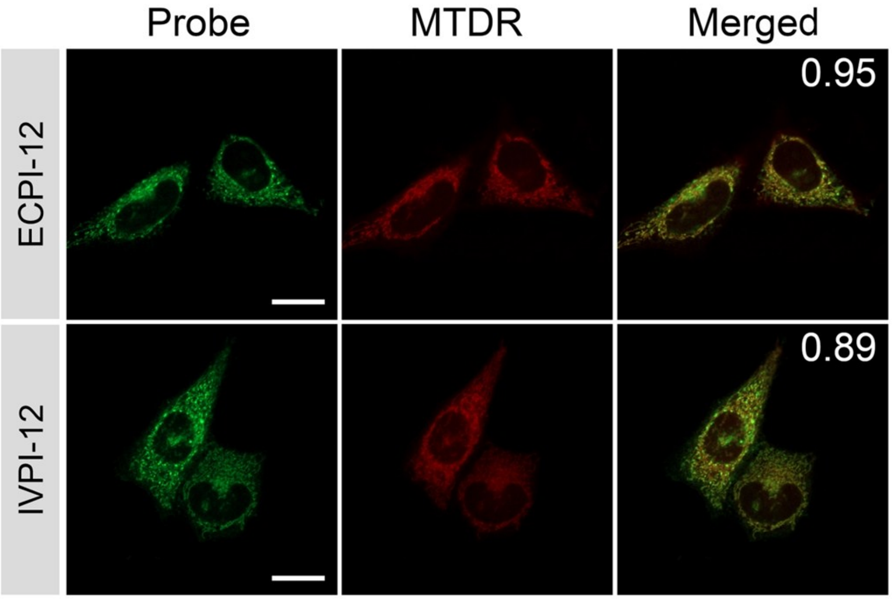

Fig. S28 Co-stain images of live HeLa cells stained with 0.2 μM ECPI-12 or IVPI-12 and 0.2 μM MTDR for 30 min, treated with 4% paraformaldehyde for 30 min, and placed for 12 h. Co-localization coefficients of ECPI-12 or IVPI-12 and MTDR were shown in the merged images. Scale bar = 20 μm.

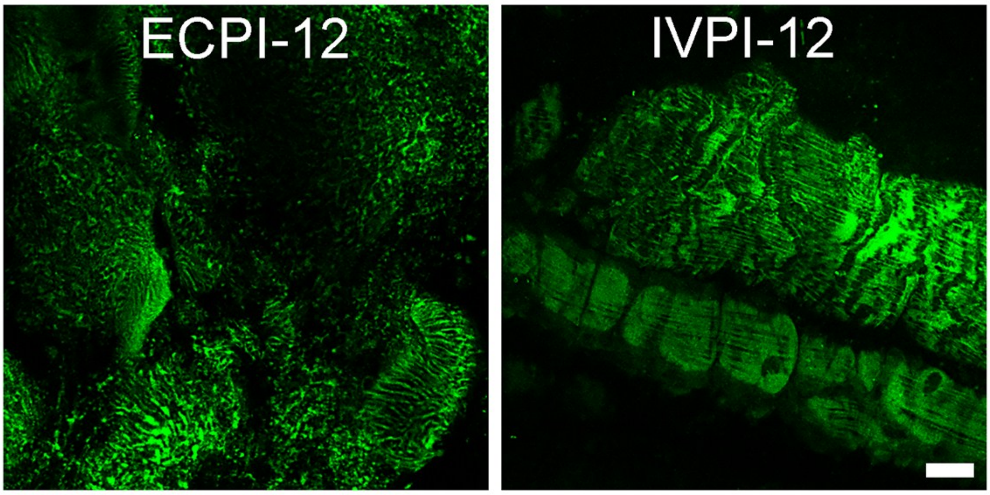

Fig. S29 *Ex vivo* two-photon microscopy images of mouse skeletal muscle tissue stained with 0.2 μM ECPI-12 and 0.2 μM IVPI-12. Excitation: 840 nm for ECPI-12 and 860 nm for IVPI-12.

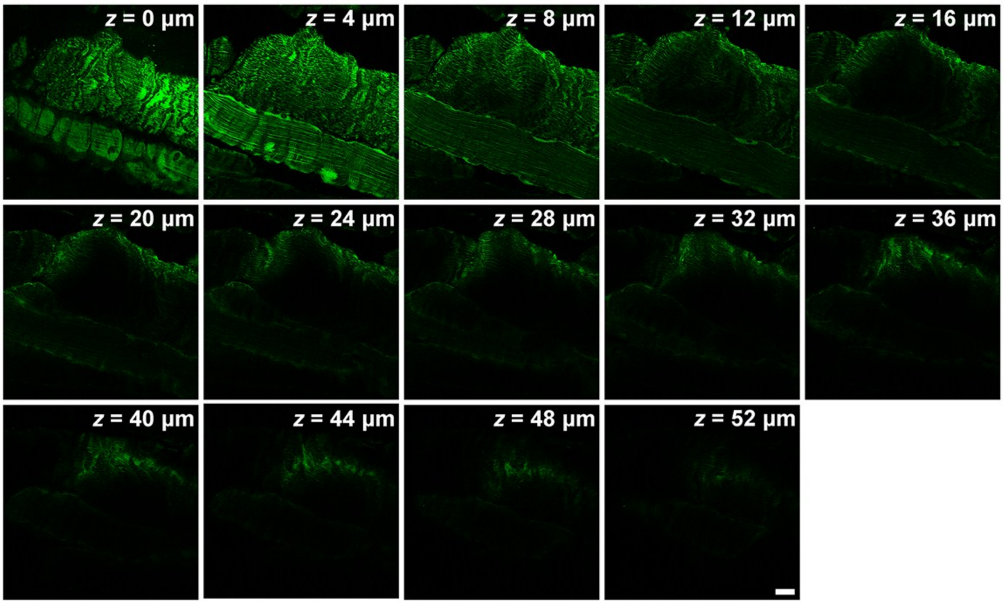

Fig. S30 *Ex vivo* two-photon (λ_{ex} = 860 nm) microscopy images of mouse skeletal muscle tissue stained with 0.2 μM IVPI-12 at different penetration depths along the *Z* axis. Scale bar = 20 μm.

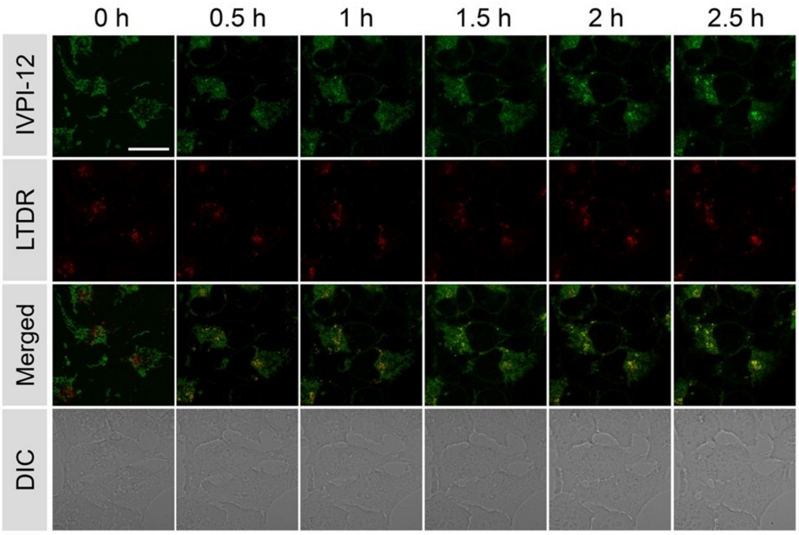

Fig. S31 Co-stain images of HeLa cells stained with 0.2 μM IVPI-12 and 0.2 μM LTDR after treatment with 10 μM CCCP and 7.5 μM pepstatin A at different time points. Scale bar = 20 μm.

Table S5 Co-localization coefficients of ECPI-12/IVPI-12 and LTDR at different CCCP-treated time points

CCCP-treated time	0 h	0.5h	1 h	1.5 h	2 h	2.5 h
ECPI-12	0.15	0.68	0.70	0.75	0.79	0.81
IVPI-12	0.25	0.68	0.72	0.74	0.79	0.82

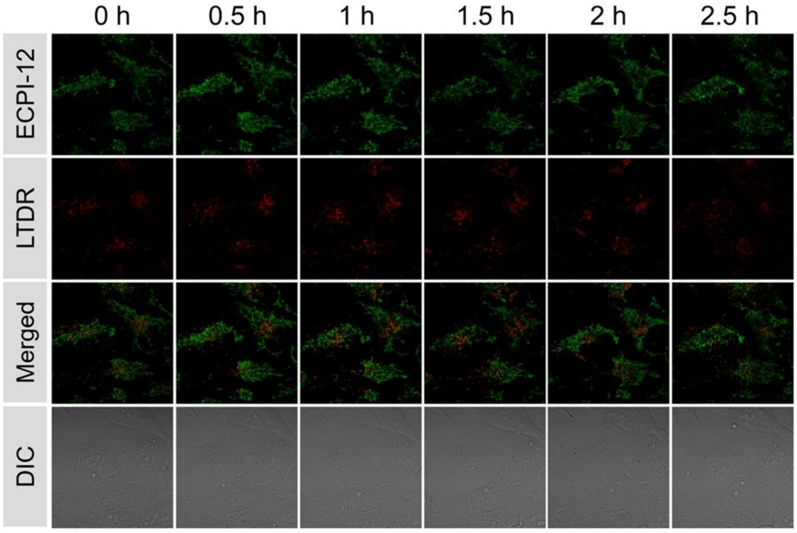

Fig. S32 Co-stain images of HeLa cells stained with 0.2 μM ECPI-12 and 0.2 μM LTDR at different time points. Scale bar = 20 μm.

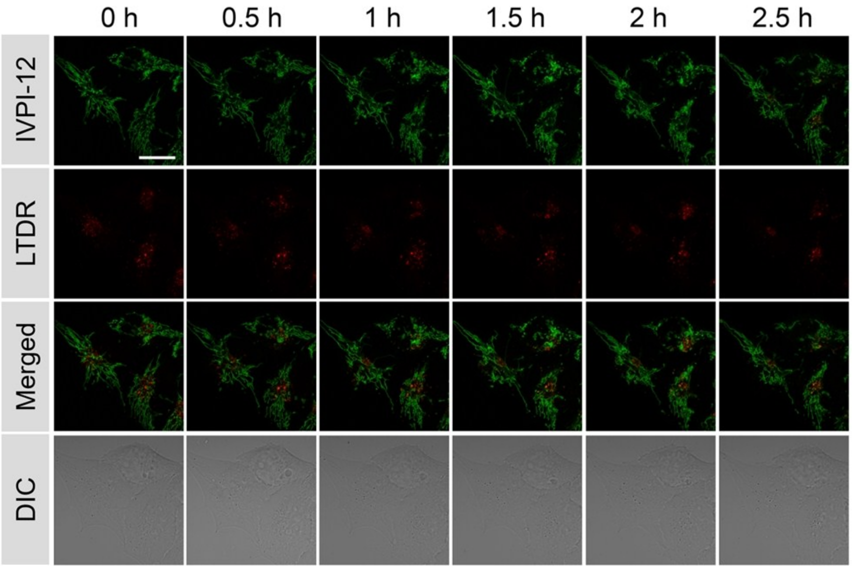

Fig. S33 Co-stain images of HeLa cells stained with 0.2 μM IVPI-12 and 0.2 μM LTDR at different time points. Scale bar = 20 μm.

Table S6 Co-localization coefficients of ECPI-12/IVPI-12 and LTDR at different time points

Time	0 h	0.5 h	1 h	1.5 h	2 h	2.5 h
ECPI-12	0.17	0.20	0.19	0.22	0.23	0.25
IVPI-12	0.12	0.19	0.18	0.20	0.22	0.25

6. Reference

[1] Z. Yang, N. Zhao, Y. Sun, F. Miao, Y. Liu, X. Liu, Y. Zhang, W. Ai, G. Song, X. Shen, X. Yu, J. Sun, W.-Y. Wong, *Chem. Commun.* **2012**, *48*, 3442-3444.

[2] R. Zhang, Y. Sun, M. Tian, G. Zhang, R. Feng, X. Li, L. Guo, X. Yu, J. Z. Sun, X. He, *Anal. Chem.* **2017**, *89*, 6575-6582.

[3] N. S. Makarov, M. Drobizhev, A. Rebane, *Opt. Express* **2008**, *16*, 4029-4047.
